# Child exposure to organophosphate and pyrethroid insecticides measured in urine, wristbands, and household dust and its implications for child health in South Africa: A panel study

**DOI:** 10.1097/EE9.0000000000000282

**Published:** 2023-12-29

**Authors:** Adriana Fernandes Veludo, Martin Röösli, Mohamed Aqiel Dalvie, Petra Stuchlík Fišerová, Roman Prokeš, Petra Přibylová, Petr Šenk, Jiří Kohoutek, Mufaro Mugari, Jana Klánová, Anke Huss, Daniel Martins Figueiredo, Hans Mol, Jonatan Dias, Céline Degrendele, Samuel Fuhrimann

**Affiliations:** a Swiss Tropical and Public Health Institute (Swiss TPH), Allschwil, Switzerland; b University of Basel, Basel, Switzerland; c Centre for Environmental and Occupational Health Research, School of Public Health, University of Cape Town, Cape Town, South Africa; d Masaryk University, Faculty of Science, RECETOX, Brno, Czech Republic; e Global Change Research Institute of the Czech Academy of Sciences, Brno, Czech Republic; f Institute for Risk Assessment Sciences, Utrecht University, Utrecht, the Netherlands; g Wageningen Food Safety Research, part of Wageningen University & Research, Wageningen, The Netherlands; h Aix-Marseille University, CNRS, LCE, Marseille, France

**Keywords:** Child exposure, Biomonitoring, Pesticide, Vulnerable populations, Endocrine disruptors

## Abstract

**Background:**

Children in agricultural areas are exposed to organophosphate (OP) and pyrethroid (PYR) insecticides. This explorative study investigated child exposure to OPs and PYRs, comparing temporal and spatial exposure variability within and among urine, wristbands, and dust samples.

**Methods:**

During spraying season 2018, 38 South African children in two agricultural areas (Grabouw/Hex River Valley) and settings (farm/village) participated in a seven-day study. Child urine and household dust samples were collected on days 1 and 7. Children and their guardians were wearing silicone wristbands for seven days. Intraclass correlation coefficients (ICCs) evaluated temporal agreements between repeated urine and dust samples, Spearman rank correlations (Rs) evaluated the correlations among matrices, and linear mixed-effect models investigated spatial exposure predictors. A risk assessment was performed using reverse dosimetry.

**Results:**

Eighteen OPs/PYRs were targeted in urine, wristbands, and dust. Levels of chlorpyrifos in dust (ICC = 0.92) and diethylphosphate biomarker in urine (ICC = 0.42) showed strong and moderate temporal agreement between day 1 and day 7, respectively. Weak agreements were observed for all others. There was mostly a weak correlation among the three matrices (Rs = −0.12 to 0.35), except for chlorpyrifos in dust and its biomarker 3,5,6-trichloro-2-pyridinol in urine (Rs = 0.44). No differences in exposure levels between living locations were observed. However, 21% of the urine biomarker levels exceeded the health-risk threshold for OP exposure.

**Conclusions:**

Observed high short-term variability in exposure levels during spraying season highlights the need for repeated sampling. The weak correlation between the exposure matrices points to different environmental and behavioral exposure pathways. Exceeding risk thresholds for OP should be further investigated.

**What this study adds**

Insecticides are widely applied for agriculture and residential use, and cumulative exposure over time may pose a possible health concern for local communities. We studied children’s organophosphate and pyrethroid insecticides exposure who are living in agricultural areas of South Africa. Therefore, we integrated multiple exposure assessment methods to understand different exposure routes. We found a high short-term exposure variability and highlighted the need for repeated sampling for accurate exposure characterization, which is particularly important for epidemiological studies. Also, our findings indicated that 21% of the children in our study exceeded the health-risk threshold for cumulative exposure to organophosphates, which should be followed up in larger, in-depth studies to understand exposure pathways and health impacts.

## Introduction

Pesticides in the chemical groups of organophosphates (OPs) and pyrethroids (PYRs) are among the most used insecticides in agriculture,^1^ for disease vector control and household pest control.^2^ Due to their modes of action and toxicity, OPs act as acetylcholinesterase (AChE) inhibitors and PYRs as sodium channel modulators, and human exposure to these groups of chemicals has been linked to several adverse health effects.^3–9^ Children and young adolescents are particularly vulnerable to pesticide exposure due to increased body burden from dermal absorption of chemicals (higher surface body area/weight ratios as compared with adults), increased respiratory rates, and fragile immune system.^10^ Studies have shown associations between early-life exposure to OPs and PYRs and impaired cognitive development,^6,11^ attention deficit hyperactivity disorder,^12,13^ or autism-related traits^14^ in children and adolescents.

Over time, cumulative exposure to OPs and PYRs occurs via many occupational and environmental exposure pathways.^15–17^ Children, particularly those living in agricultural areas, can be exposed during their daily-life activities (e.g., playing in previously sprayed fields or when partaking in agricultural activities) or ingesting contaminated food or water.^18–21^ Children can additionally be exposed at home via dust contaminated with pesticides that accumulate in carpets or other surfaces due to indoor pesticide use,^22^ spray drift from nearby farms,^23,24^ or take-home pesticides from household members’ shoes or clothes.^25^

Although multiple exposure studies rely on self-reported data to understand determinants of exposure,^2,19,21,26^ there are growing efforts to use different methods as tools to investigate potential exposure sources (e.g., combining biomonitoring with point-of-contact or scenario-based assessments).^27–32^ Biomonitoring is used to measure biological indicators (i.e., biomarkers) after exposure has occurred.^33,34^ Urinary biomarkers can be indicators of exposure to specific active ingredients (e.g., 3,5,6-trichloro-2-pyridinol [TCPy], the biomarker of chlorpyrifos and chlorpyrifos-methyl) or to chemical groups (e.g., dialkyl phosphate metabolites [DAPs] reflect exposure to multiple OPs).^15,34,35^ Nevertheless, these are rapidly metabolized and excreted from the body after exposure (half-lives ranging from 2 to 41 hours, depending on the pesticide and the exposure route). Moreover, their levels can vary greatly between and within individuals, and there is still a lack of understanding of how urinary levels correlate with external exposure matrices.^36^ Point-of-contact exposure assessments use personal monitoring equipment that records cumulative individual exposure levels over time. Silicone wristbands have been increasingly used as personal passive samplers as they can capture multiple organic pollutants such as pesticides. They are low-cost and noninvasive tools that reflect dermal and inhalation exposure routes in different microenvironments (i.e., indoor and outdoor).^37–41^ Finally, scenario-based assessments are used to determine pesticide exposure in specific environments.^42,43^ For example, home exposure to OPs and PYRs via ingestion of dust particles has been assessed using measured pesticide levels in household dust^44^ and information on daily dust ingestion rates (age-dependent modeled values^45,46^), body weight, and time spent at home.^47,48^

Such studies that integrate multiple methods to assess exposure are largely lacking in low- and middle-income countries, where pesticides are heavily applied in agriculture and for household pest control. South Africa is the heaviest pesticide user in sub-Saharan Africa, with more than 20000 tonnes of pesticides used in agriculture yearly.^49^ This results from an intensification of the cropping systems and expansion of agricultural areas.^50^ More than 50 OPs and PYRs active ingredients are registered for agriculture or household insect control in South Africa. Further, there are reports that some are decanted and illegally sold on street markets for household pest control.^22,51^

Hence, in this article, we aimed to tackle the gaps in the literature by measuring children’s exposure to OPs and PYRs using different exposure assessment methods (urinary biomarkers, silicone wristbands, and household dust) and to determine their spatial and temporal variability during the 1 week in the spraying season in two agricultural areas of South Africa. The four specific objectives were (1) to study the temporal agreement between repeated urine and dust measurements, (2) to investigate the correlation among urinary biomarkers, silicone wristbands, and household dust levels, (3) to understand if individual exposure mixtures cluster according to their living locations, and (4) to estimate the health risk of exposure using repeated urinary biomarker levels.

## Methods

### Study area

The present study was conducted in the Western Cape, South Africa region, which covers approximately 13 million hectares, of which two million are dedicated to agricultural use.^52^ Within the Western Cape, two rural study areas were selected based on their different crop profiles: Hex River Valley (33°28′34.7″S19°39′51.9″E), with table grapes representing 98% of the agricultural land use, and Grabouw (34°09′16.8″S18°59′56.7″E), where pome fruits represent 81% of the agricultural land use.

### Study design

This panel study is part of the ongoing “Child Health Agricultural Pesticide Cohort Study in South Africa” (CapSA) project, which aims to determine the association between agricultural pesticide exposure and its potential health effects on 1000 children.^19,^^53^

**Figure 1.**
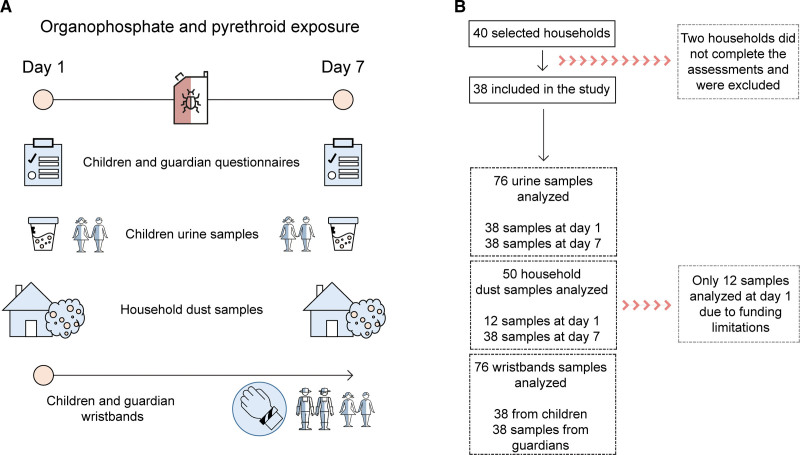
Study design (A) and consort flow chart showing the participant and sample selection (B).

Out of the CapSA cohort, 40 households were purposely selected alongside the main urine sampling round of all 1000 children to guarantee equal participant numbers living in two agricultural areas (Hex River Valley and Grabouw, Figure 1A). Within each area, half of the households were located on farms (within 50 m of agricultural land use) and half in nearby villages (at least 0.5 km from the closest agricultural land use).^37^ These two household settings were a priori selected to account for possible spatial differences in pesticide concentrations within each area.

### Data collection

The sampling campaign was conducted in 2018 for over 7 days, during the main pesticide spraying season in Hex River Valley (between the 23 and 29 October) and in Grabouw (between the 31 October and the 6 November). Out of the 40 selected households, two did not complete the assessments (i.e., collection of urine and wristbands) and/or wished not to participate, resulting in 38 households included in the study (Figure 1B). In total, 76 wristbands (38 children and their respective guardians), 76 urine samples (38 on days 1 and 7), and 50 dust samples (12 on day 1 and 38 on day 7) were analyzed. Unfortunately, due to logistical constraints, only a subsample of the household dust samples could be analyzed on day 1.

A trained interviewer gave children and guardians separate structured questionnaires developed for the CapSA study^53^ (Table 1). The children’s questionnaire was administered after each urine sampling at their respective schools. Children’s questionnaires included questions on sociodemographic characteristics (e.g., sex, age, and education), activities on the farm (i.e., picking fruits or helping with other tasks), and leisure activities (i.e., swimming in ponds or spending time in agricultural fields). The guardians were interviewed during home visits when the dust samples were collected. The guardian’s questionnaire included sociodemographic characteristics, occupation, and household pesticide use. These questionnaires were only administered on day 1 as it was anticipated that there would be little variations between day 1 and day 7. Both child and guardian interviews were conducted in the participant’s mother tongue (i.e., English, Afrikaans, or Xhosa), and the questionnaires were back translated to English.

**Table 1.** Sociodemographic and occupational characteristics of participating children and their guardians stratified by study area

		Total, n (%)	Grabouw, n (%)	Hex River Valley, n (%)
		38 (100)	19 (100)	19 (100)
Place of living	Farm	20 (52.6)	10 (52.6)	10 (52.6)
	Village	18 (47.4)	9 (47.4)	9 (47.4)
Child				
Sex	Female	16 (42.1)	7 (36.8)	9 (47.4)
	Male	22 (57.9)	12 (63.2)	10 (52.6)
Age in years (median; IQR)	Female	(12; 11–13)	(12; 11–12)	(12; 11–13)
	Male	(12; 11–12)	(12; 10–13)	(12; 11–12)
BMI in kg/m^2^ (median; IQR)^a^	Female	(17.2; 15.3–18.6)	(17.1; 15.5–20.1)	(17.6; 14.5–18.1)
	Male	(17.2; 15.4–20.6)	(18.8; 16.5–21.5)	(15.2; 14.6–16.3)
Observed spraying activities^b^ 7 days prior the measurement week During the measurement week	Yes Yes	32 (84.2) 30 (78.9)	16 (84.2) 16 (84.2)	16 (84.2) 14 (73.7)
Swum in a pond or river^b^ 7 days prior the measurement week During the measurement week	Yes Yes	18 (47.4) 18 (47.4)	8 (42.1) 9 (47.4)	10 (52.6) 9 (47.4)
Played in agriculture fields treated with pesticides^b^ 7 days prior the measurement week During the measurement week	Yes Yes	13 (34.2) 10 (26.3)	6 (31.6) 5 (26.3)	7 (36.8) 5 (26.3)
Helped to pick fruits from fields^b^ 7 days prior the measurement week During the measurement week	Yes Yes	17 (44.7) 14 (36.8)	4 (21.1) 7 (36.8)	13 (68.4) 7 (36.8)
Engaged in pesticide-handling activities^b^ 7 days prior the measurement week During the measurement week	Yes Yes	15 (39.5) 21 (55.3)	10 (52.6) 10 (52.6)	5 (26.3) 11 (57.9)
Guardian^c^				
Sex	Female	35 (92.1)	18 (94.7)	17 (89.5)
	Male	3 (7.9)	1 (5.3)	2 (10.5)
Interviewed guardian works on farm^d^	Yes	14 (36.8)	4 (21.1)	10 (52.6)
Any household member working on a farm^d^	Yes	25 (65.8)	10 (52.6)	15 (78.9)
Any household member washes working clothes at home^d^	Yes	21 (55.3)	6 (31.6)	15 (78.9)
Any pesticide sprayed at home	No	7 (18.4)	6 (78.9)	1 (5.3)
	Last year	10 (26.3)	6 (31.6)	4 (21.1)
	Last week^d^	21 (55.3)	7 (36.8)	14 (73.7)

a Two children did not provide information on their height and weight; therefore, BMI was calculated based on 36 observations (19 in Grabouw and 17 Hex River Valley).

b Observed spraying: 63% and 66% of those that answered “yes” prior and during measurement week, respectively, live in farms; swum in pond/river: 72% and 56% of those that answered “yes” prior and during measurement week, respectively, live in farms; play in sprayed fields: 69% and 80% of those that answered “yes” prior and during measurement week, respectively, live in farms; helped picking fruits from fields: 71% and 64% of those that answered “yes” prior and during measurement week, respectively, live in farms; and engaged in pesticide-handling activities: 80% and 81% of those that answered “yes” prior and during measurement week, respectively, live in farms.

c Questionnaire data collected on day 1.

d Guardian works in farm: 79% of those that answered “yes” live in farms; household member works in farm: 76% of those that answered “yes” live in farms; wash work clothes at home: 71% of those that answered “yes” live in farms; and sprayed pesticides at home the week prior the measurement week: 33% of those that answered “yes” live in farms.

#### Urine sample collection

Within the measurement week, spot urine samples were collected from each child twice. The first urine samples were collected at the beginning of the study (day 1), whereas the last samples were gathered on the last day of the study (day 7). The urine samples were collected during a morning school break at the participant’s respective school (these do not represent first-morning voids). Urine plastic containers were given to each child and collected after they were filled with urine, according to the sampling protocol. These were subsequently collected into 8-mL plastic vials and kept in a freezer until they were shipped to the accredited trace analytical laboratories, as described by Fišerová et al.^54^ Urine samples were sent to RECETOX, Masaryk University, Czech Republic, for further analysis.

#### Wristband collection

The selected children and their guardians were further asked to wear a wristband for the whole study duration (also during showering and sleeping). Every morning, children were examined at school to confirm if they were wearing their wristbands. For good compliance, the children were reminded daily about compliance at school. On day 7, the wristbands were collected, stored, and shipped in a cool box at 0 °C to Wageningen Food Safety Research (WFSR), Wageningen University, the Netherlands, for analysis.^37^

#### Household dust collection

Repeated dust samples were collected from each household, once on day 1 and once on day 7. The dust samples were taken from the children’s bedroom using a stainless-steel inlet equipped with a preseparation mesh connected to a vacuum cleaner, as previously reported by Degrendele et al.^44^ Following collection, the dust samples were packed in two layers of aluminum foil and sealed in a plastic bag. The samples in a cooler box were shipped to RECETOX laboratory, Masaryk University, the Czech Republic and stored at −18 °C until processing.

### Sample preparation and analysis

The detailed sample preparation, extraction, and analysis (including quality assurance and quality control) have been previously described for the urine,^54^ wristbands,^37^ and household dust^44^ samples used in this study. A brief description of the respective methods is provided below. The selection of pesticides to be analyzed was motivated by four factors: (1) their current use in South Africa for agricultural purposes;^55^ (2) their past use in agriculture;^56,57^ (3) their potential use at household level;^58^ and (4) the selection was limited to the analytical capacity of the selected laboratory method.

#### Urine sample preparation and analysis

Overall, 10 OPs and three PYRs urinary biomarkers were targeted. OPs biomarkers included five unspecific DAP metabolites (diethylphosphate [DEP], diethylthiophosphate [DETP], diethyldithiophosphate [DEDTP], dimethylphosphate [DMP], and dimethylthiophosphate [DMTP]) and five more specific metabolites (malathion dicarboxylic acid [MDA], *p*-nitrophenol [PNP], 3-chloro-4-methylumbelliferone [CMHC], 3,5,6-trichloro-2-pyridinol, and 2-isopropyl-4-methyl-6-hydroxypyrimidine [IMPy]). PYRs biomarkers included two unspecific metabolites (3-phenoxybenzoic acid [3-PBA] and *cis*-/*trans*-3-(2,2-dichlorovinyl)-2,2-dimethyl cyclopropane-carboxylic acid [DCCA]) and one specific metabolite (4-fluoro-3-phenoxybenzoic acid [4F3-PBA]). The extraction of four DAP metabolites (i.e., DEP, DETP, DMP, and DMTP) was done by applying the QuEChERS-based method. The remaining pesticide metabolites were extracted via solid-phase extraction. Pesticide metabolites and creatinine analysis were performed using high-performance liquid chromatography coupled to mass spectrometry (HPLC-MS).^53^

The urinary biomarkers and creatinine concentrations are in Table S1; http://links.lww.com/EE/A252. To adjust for urinary dilution, the following equation was used:


(1)

#### Wristband precleaning, extraction, and analysis

The wristbands were precleaned and shipped to South Africa, where they were placed in individual zip-lock mylar bags (DS M&T Inc., Fontana, California). After the sampling campaign, the wristbands were extracted and analyzed using a gas chromatography–tandem mass spectrometry system.^37^ Overall, five OPs (chlorpyrifos, diazinon, malathion, prothiofos, and dimethoate) and three PYRs (cypermethrin, deltamethrin, and λ-cyhalothrin) were targeted in wristbands.

#### Household dust sample preparation and analysis

Three OPs (chlorpyrifos, diazinon, and malathion) were targeted in the dust. The samples were extracted with methanol using an ultrasonic bath for about 1 hour and further analyzed using HPLC-MS.^44^ Unfortunately, no PYRs were analyzed in dust samples due to the limited analytical capacity of the selected laboratory.

### Data analysis

Descriptive statistics provided information on the quantification frequency, median (interquartile range [IQR]), and maximum levels of OPs and PYRs above the quantification limit in each of the three matrices (creatinine-corrected biomarkers, wristbands, and dust) (Table 2). The raw data for each matrix can be found in Tables S1–S3; http://links.lww.com/EE/A252. All analyses were performed using R software (Foundation for Statistical Computing, version 3.5.3, RStudio Version 1.1.4).

**Table 2.** Quantification frequency and levels of the targeted OP and PYR insecticides in urine, wristbands, and household dust samples

Urine samples (µg/g creatinine)^a^											
			Total (n = 76)			Day 1 (n = 38)			Day 7 (n = 38)		
Biomarker	Group	Active ingredient	Detects, n (%)	Median (IQR)	Max	Detects, n (%)	Median (IQR)	Max	Detects, n (%)	Median (IQR)	Max
3-PBA	PYR	Unspecific^b^	75 (98.7)	1.0 (0.68–1.5)	9.6	37 (97.4)	1.0 (0.7–1.6)	9.6	38 (100)	1 (0.7–1.3)	5.1
4F3-PBA	PYR	Cyfluthrin^c^	18 (23.7)	1.1 (1.0–1.4)	4	18 (47.4)	1.1 (1.0–1.4)	4	Not detected		
CMHC	OP	Coumaphos	2 (2.6)	0.04 (0.04–0.04)	0.04	Not detected			2 (5.3)	0.04 (0.04–0.04)	0.04
DCCA	PYR	Unspecific^d^	76 (100)	0.8 (0.6–1.1)	12.9	38 (100)	0.8 (0.6–1.2)	12.9	38 (100)	0.6 (0.6–0.9)	3
DEP	OP	Unspecific^e^	70 (92.1)	2.5 (1.8–4.9)	22.8	35 (92.1)	2.6 (2.1–5.4)	22.8	35 (92.1)	2.3 (1.7–4.4)	22.6
DETP	OP	Unspecific^e^	76 (100)	0.8 (0.5–1.1)	15.1	38 (100)	0.8 (0.6–1.1)	15	38 (100)	0.8 (0.4–1.3)	15.1
DMP	OP	Unspecific^e^	74 (97.4)	13.5 (8.7–22.1)	112.4	36 (94.7)	11.1 (8.6–16.5)	63.2	38 (100)	14.7 (9.3–24.4)	112.4
DMTP	OP	Unspecific^e^	76 (100)	2.0 (1.5–3.4)	19.2	38 (100)	2.3 (1.5–3.2)	19.2	38 (100)	2.4 (1.5–3.6)	17.1
IMPy	OP	Diazinon	62 (81.6)	0.41 (0.2–1.2)	21.5	36 (94.7)	0.6 (0.2–2.3)	21.5	26 (68.4)	0.3 (0.1–0.6)	15.8
TCPy	OP	Chlorpyrifos^f^	74 (94.7)	1.0 (0.4–2.6)	113	38 (100)	1.9 (0.8–3.9)	113	34 (89.5)	0.4 (0.1–1.0)	6.8
PNP	OP	Parathion^g^	54 (71.1)	0.3 (0.1–0.6)	3.5	30 (78.9)	0.2 (0.1–0.5)	3.47	24 (63.2)	0.5 (0.2–0.7)	1.1
DEDTP	OP	Unspecific^e^	Not detected								
MDA	OP	Malathion	Not detected								
Wristband samples (ng/g wristband)											
			Total (n = 76)			Children (n = 38)			Guardians (n = 38)		
Active ingredient	Group		Detect, n (%)	Median (IQR)	Max	Detects, n (%)	Median (IQR)	Max	Detects, n (%)	Median (IQR)	Max
Deltamethrin	PYR		69 (90.8)	3.8 (2.5–5.6)	352.3	36 (94.7)	4.0 (3.2–6.0)	107	33 (86.8)	3.1 (2.0–4.9)	352.3
Chlorpyrifos	OP		61 (80.3)	62.7 (25.7–158.0)	658.5	29 (76.3)	89.8 (43.2–273.6)	658.5	32 (84.2)	29.5 (14.0–88.3)	386.1
Cypermethrin	PYR		43 (56.6)	19.2 (9.9–32.0)	88.6	24 (63.2)	20.2 (12.5–38.4)	88.6	19 (50)	15.9 (6.2–26.8)	45.6
Diazinon	OP		35 (46.1)	3.0 (1.9–8.1)	98.4	22 (57.9)	2.9 (1.8–6.3)	98.4	23 (34.2)	3.0 (1.9–11.1)	80.5
Prothiofos	OP		35 (46.1)	26.4 (10.0–55.8)	582.6	20 (52.6)	36.6 (15.1–71.2)	582.6	25 (39.5)	14.5 (5.0–41.5)	96.5
Malathion	OP		17 (22.4)	2.1 (1.4–2.8)	14.3	10 (26.3)	2.5 (1.5–2.7)	6.3	7 (18.4)	1.9 (1.3–5.3)	14.3
Dimethoate	OP		Not detected								
λ-cyhalothrin	PYR		Not detected								
Household dust samples (ng/g dust)											
			Total (n = 50)			Day 1 (n = 12)			Day 7 (n = 38)		
Active ingredient	Group		Detects, n (%)	Median (IQR)	Max	Detects, n (%)	Median (IQR)	Max	Detects, n (%)	Median (IQR)	Max
Chlorpyrifos	OP		48 (96.0)	367.8 (152.0–1009.5)	19528.3	12 (100)	381.1 (215.7–616.9)	3800	36 (94.7)	367.8 (131.6–1075.0)	19528.3
Diazinon	OP		36 (72.0)	15.4 (7.2–39.3)	2209.5	11 (91.7)	10.9 (5.8–19.8)	1396	25 (65.8)	17.8 (10.9–54.5)	2209.5
Malathion	OP		2 (4.0)	96.7 (70.3–123.1)	149.5	Not detected			2 (5.3)	96.7 (70.3–123.1)	149.5

a Unadjusted urine levels are available in Table S1; http://links.lww.com/EE/A252.

b Biomarker of cypermethrin, deltamethrin, etofenprox, fenpropathrin, fenvalerate, esfenvalerate, λ-cyhalothrin, permethrin, and τ-fluvalinate.

c Also β-cyfluthrin.

d Biomarker of cyfluthrin, cypermethrin, and permethrin.

e An extended list of parent compounds can be found in the literature.^59^

f Also chlorpyrifos-methyl.

g Also methyl-parathion.

#### Temporal agreement between repeated urine and dust measurements

Spearman’s rank correlations (Rs) and intraclass correlation coefficient (ICC) were calculated for urinary biomarkers and dust pesticides to understand the temporal correlation and agreement between the repeated samples (i.e., on day 1 and day 7). Rs values between ±0 and ±0.3 indicate weak correlations, values between ±0.4 and ±0.6 indicate moderate correlations, values between ±0.7 and ±0.9 indicate strong correlations, and any value equal to ±1 indicates a perfect correlation.^59^ ICC values below 0.5 indicate poor temporal agreement, values between 0.5 and 0.75 indicate moderate agreement, values between 0.75 and 0.9 indicate good agreement, and any value above 0.9 indicates excellent agreement.^60^ The urinary biomarkers 4F3-PBA and malathion in dust were never quantified on day 7 and day 1, respectively, and were dropped from the analysis.

#### Correlation among urinary biomarkers, wristbands, and household dust levels

A correlation matrix using pairwise Spearman’s rank correlations was created to understand the levels of correlation between compounds measured on day 7 in the different matrices (i.e., urinary biomarkers, wristbands, and household dust). Measurements collected on day 1 were not included in the matrix due to incomplete data for dust samples. However, this information can be found in Figure S1; http://links.lww.com/EE/A252 and Table S4; http://links.lww.com/EE/A252. Only the compounds with representation in at least two matrices were included (i.e., PNP and 4F3-PBA biomarkers were excluded due to no representation in wristbands or dust; Table 2). To deal with the different unit levels inherent to each matrix, the concentrations were first log transformed and standardized to a mean of zero and a standard deviation of one.

#### Spatial clustering of exposure mixtures

To visualize the clustering of individuals exposed to different insecticide mixtures, heatmaps were created showing the log-standardized levels of child urinary biomarkers (day 1 and day 7) and child and guardian wristbands and household dust (day 1 and day 7). The allocated household IDs were hierarchically clustered using the Spearman’s rank correlation as a distance measure to understand how the exposure levels correlated between participants living in different areas and household settings.

Multivariable linear mixed-effect (LME) models, using household IDs as a random effect, were performed to study potential exposure predictors (study area [Grabouw and Hex River Valley] and household setting [farm and village]) while correcting for the sex of the participant (with exception to the dust models). Even though age is an important confounder, the models were not corrected for this, given the little variability in age between participants (Table 1) and the small sample size of the study population. A first model (hereafter, the overall model) treated all pesticide values (regardless of the matrix) as repeated measurements. Similarly to what was performed in *Correlation among urinary biomarkers, wristbands, and household dust levels*, the data was log transformed and standardized to account for the different unit levels inherent to each matrix. Subsequent models were first stratified per matrix (i.e., urine, dust, and wristbands) and then stratified per pesticide within the matrices. This allows us to understand whether overall exposure to pesticides is strongly dictated by the living location of the participant or whether this is highly variable according to the exposure route or the specific pesticide analyzed. A *P* value <0.05 was considered statistically significant.

#### Health-risk assessment using the urinary biomonitoring levels

A reverse dosimetry approach was used to evaluate the exposure risk to the specific OPs (chlorpyrifos, diazinon, and parathion) and PYRs (cypermethrin and deltamethrin) insecticides.

The repeated urinary biomarker measurements were used to perform a rough estimate of the daily intake (EDI, µg/kg/d) of pesticides as follows:^61–63^


(2)

where *C*_U_ represents the urinary concentration of the metabolite (µg/L) (values not corrected for creatinine, Table S1; http://links.lww.com/EE/A252), *V*_24_ the child’s daily urinary excretion volume (L/d), MW_P_ the molecular weight of the active ingredient (g/mol), *F*_UE_ the urinary excretion factor of each active ingredient (unitless) (Table S5; http://links.lww.com/EE/A252), BW the child’s body weight (kg), and MW_M_ is the molecular weight of the biomarker (g/mol). The mean values of *V*_24_ were taken from the literature according to each child’s age group.^64^ For the children that did not provide their BW (two children), we took the mean BW value from children of the same age and sex. Where available, the EDI of each pesticide was calculated using the specific urinary biomarker for that pesticide. For chlorpyrifos, the EDI was calculated using both the specific biomarker (TCPy) and the ∑DEPs (i.e., DEP + DETP [DMP and DMTP only reflect exposure to chlorpyrifos-methyl^65]^) for comparison between approaches. The urinary levels of DCCA and 3-PBA were considered for cypermethrin and deltamethrin, respectively (no specific biomarkers are available). The parameters used to calculate the EDI are shown in Table S5; http://links.lww.com/EE/A252. Although biomarkers can be detected in their form in environmental samples,^29,66^ here we assumed that the presence of the biomarkers in urine was exclusively derived from exposure to the active ingredient and not to the metabolite itself. After calculating the EDI values, we estimated the hazard quotient (HQ) by dividing the EDI by the acceptable daily intake (ADI), a toxicological reference value:


(3)

A value below one indicates a low risk of exposure to that specific pesticide. The ADIs used in this study were those proposed by the European Food Safety Authority (EFSA).^67,68^ Finally, for each group of pesticides with the same mode of action (MoA), the hazard index (HI) was calculated to account for the cumulative risk of exposure to a specific group of pesticides:^69^


(4)

The HI was calculated separately for OPs (AChE inhibitors) and PYRs (sodium channel modulators) due to their different modes of action. For a higher specificity in the HI for OPs, the HQ for chlorpyrifos using TCPy was preferred over the ∑DEPs. A value above one indicates a high risk associated with exposure to OPs or PYRs. The median (IQR), 95th percentile, and maximum EDI, HQ, and HI were calculated for each pesticide or pesticide group on day 1 and day 7 (Table S6; http://links.lww.com/EE/A252).

#### Handling data below the limit of detection

For pesticides and biomarkers showing at least 40% of data above limit of detection (LOD) (Table 2), measurements below LOD were imputed using a maximum likelihood estimation approach, taking the area and household setting as predictors. Imputations were achieved using bootstrap randomly selected values from a log-normal estimated parameter distribution.^70^ The imputed data was used for ICC calculations, LME models, and health-risk assessment. Pesticides and biomarkers with less than 40% of data above LOD were dropped from these analyses.

### Ethical approval

Written informed consent was obtained from each guardian and children assented to participate in the study. The study received ethical clearance from the University of Cape Town’s Research Ethics Committee (HREC 637/2018).

## Results

### Demographics of the study population

The sociodemographic and occupational characteristics of the participating children and their guardians are presented in Table 1. The guardian questionnaires on day 7 were not answered appropriately; therefore, only data from day 1 is shown.

The median age of the 38 children participating in the study was 12 years (IQR, 11–13 years old). Slightly more than half of the participants were boys (58%) and lived on a farm (53%). The median body mass index (BMI) for both boys and girls was 17 kg/m^2^ (IQR, 15.3–18.6 and 16.5–21.5 kg/m^2^, respectively). Most children reported having observed spraying events in surrounding farms prior and during the measurement week (84% and 79%, respectively). Almost half of the children (47%) reported having swum in a pond/river or having helped picking fruits from agriculture fields 7 days before the measurement week. More than half of the children (55%) reported engaging in pesticide-handling activities during the measurement week (including assisting in pesticide storage or helping with spraying, mixing, or loading). It was also observed that the majority of the children that have reported engaging in activities linked with pesticide exposure before and during the measurement week (i.e., swimming in ponds/rivers, playing in previously sprayed fields, picking fruits from agriculture fields, and engaging in pesticide-handling activities) live in farms.

Of the 38 participating guardians, the majority were females (92%) and reported that at least one of the household members has a farm-related job (66%). More than half (55%) reported washing their work clothes at home and having sprayed pesticides in the household the week before the measurement week. It was observed that the majority of the guardians having reported working on a farm (79%), having any other household member working on a farm (76%), and washing work clothes at home (71%) also live on farms. On the other hand, most of those reporting to have sprayed pesticides at home before the measurement week live in villages (67%).

### OPs and PYRs levels in urine, wristbands, and dust and temporal agreement between repeated measurements

Across the analyzed matrices, a total of 18 different OPs and PYRs were targeted (Table 2), of which 15 were detected at least once.

In urine, DETP, DMTP, and DCCA were detected in all 76 urine samples (100%), followed by 3-PBA, DMP, TCPy, and DEP, which were detected in more than 90% of the samples (Table 2). The DAP metabolites were generally present in higher concentrations than the remaining OPs and PYRs metabolites, with DMP detected the highest (median, 13.4 µg/g creatinine; IQR, 8.7–22.1 µg/g creatinine).

In the 76 wristband samples, deltamethrin was detected most often (90.8%), followed by chlorpyrifos (80.3%) and cypermethrin (56.6%), with chlorpyrifos being the insecticide detected in higher concentrations (median, 61.1 ng/g wristband; IQR, 25.7–158.0 ng/g wristband).

Chlorpyrifos was also the insecticide detected in higher concentrations in the 50 dust samples analyzed (median, 367.8 ng/g dust; IQR, 152.0–1009.5 ng/g dust) and the most frequently detected (96%). Diazinon was detected in 72% of the dust samples (median, 15.4 ng/g dust; IQR, 7.2–39.3 ng/g dust), whereas malathion was only detected in 4% of the samples (median, 96.7 ng/g dust; IQR, 70.3–123.1 ng/g dust). No PYR insecticides were targeted in household dust.

**Figure 2.**
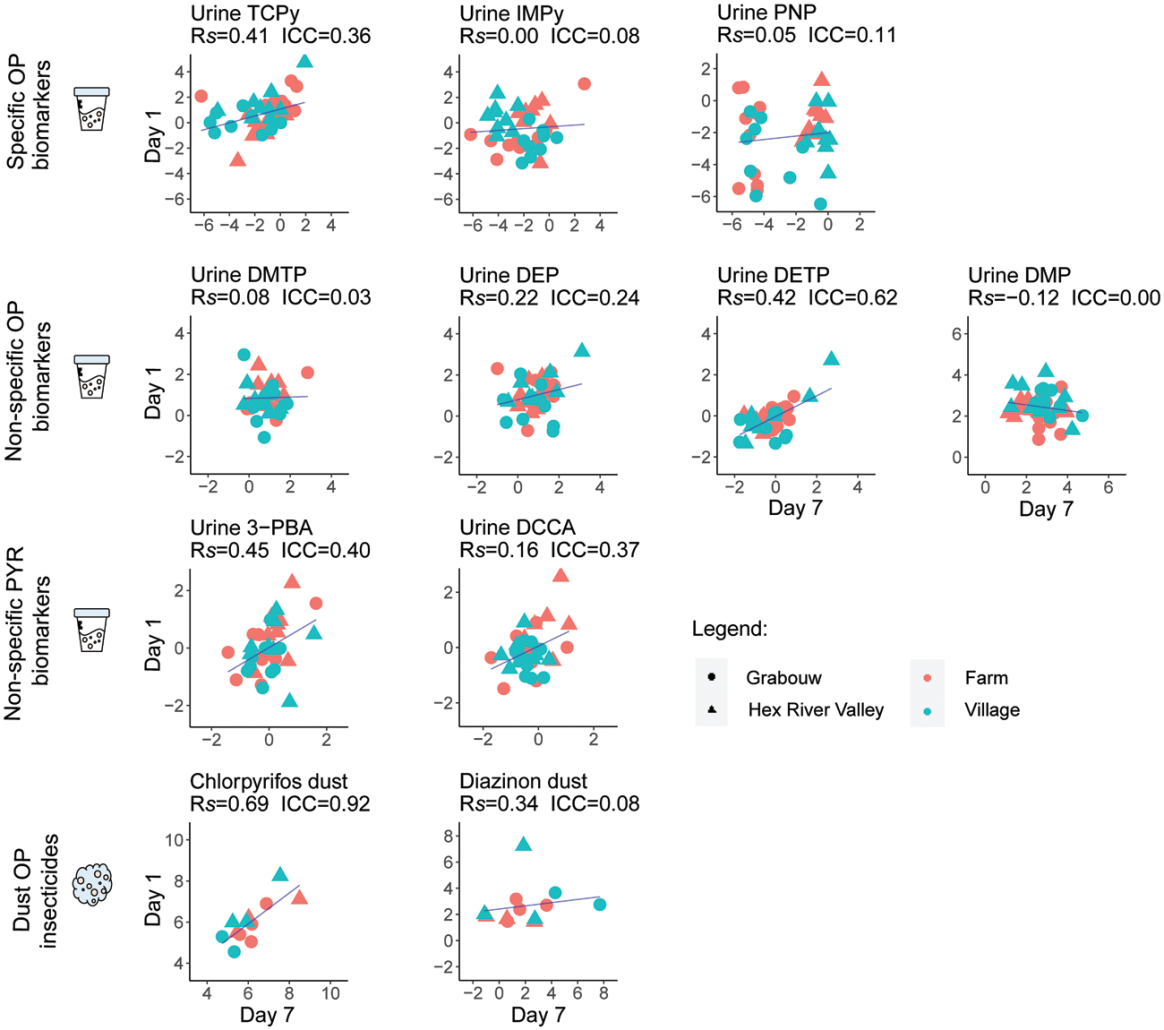
Scatter plots showing log concentrations of urinary biomarkers (µg/g creatinine) and household dust (ng/g dust) for day 1 and day 7. Spearman’s rank correlations (Rs) and ICCs between repeated measurements are also presented.

The Spearman’s rank correlations and the ICCs calculated to understand the temporal agreement between repeated measurements are presented in Figure 2. For the majority of urinary biomarkers analyzed, weak correlations and agreements were observed between levels measured on day 1 and day 7 (Rs, −0.12 to 0.22; ICC, 0.00–0.40). Nonetheless, moderate positive correlations were observed for TCPy (Rs, 0.41), DETP (Rs, 0.42), and 3-PBA (0.45), with DETP showing also a moderate agreement between repeated samples (ICC, 0.62). As to the dust samples, a strong positive correlation (Rs, 0.69) and excellent agreement (ICC, 0.93) were observed between chlorpyrifos levels measured on both days. The opposite was observed for diazinon levels in dust (Rs, 0.34; ICC, 0.15).

### Correlation within and between insecticide levels measured in urine, wristbands, and dust

**Figure 3.**
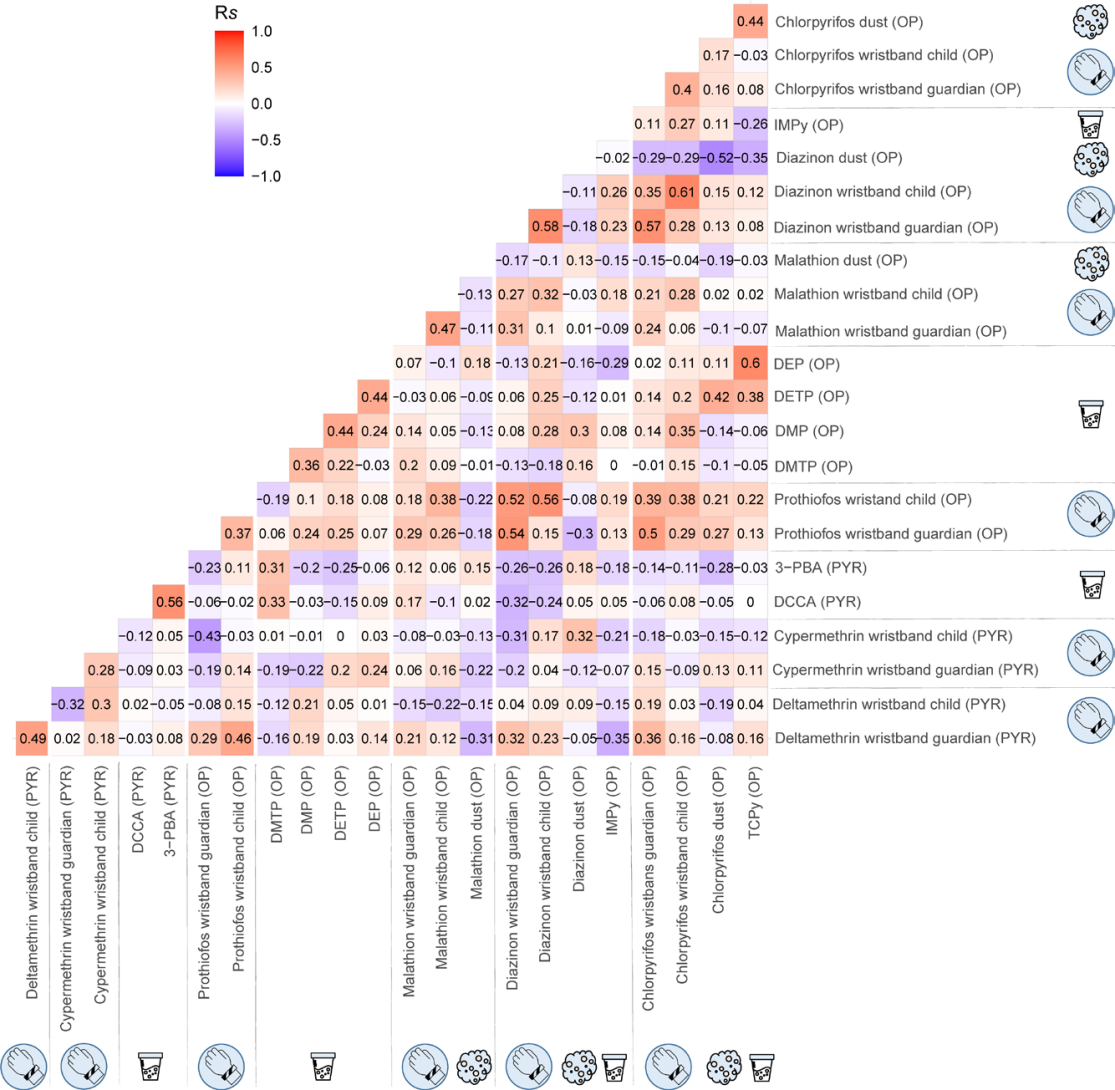
Correlation matrix showing the Spearman’s rank correlations (Rs) between the levels of organophosphates (OPs) and pyrethroids (PYRs) in urine, wristbands, and dust on day 7.

The correlation coefficients between compounds measured on day 7 in the different matrices (i.e., urinary biomarkers, wristbands, and household dust) are presented in Figure 3, whereas the respective *P* values can be found in Table S4; http://links.lww.com/EE/A252. A moderate correlation was observed for urinary biomarkers between TCPy and DEP (Rs, 0.60) and between the PYRs metabolites, that is, 3-PBA and DCCA (Rs, 0.56). A weak-to-moderate correlation was observed between pesticides measured in children’s (Rs, 0.28–0.61) and guardians’ (Rs, 0.02–0.57) wristbands. The children's wristbands were weak to moderately correlated to their guardian’s wristbands, with the strongest pair correlation observed for diazinon (Rs, 0.58). In dust, a moderate negative correlation was observed between chlorpyrifos and diazinon (Rs, −0.52) (Figure 3, Table S4; http://links.lww.com/EE/A252). In all analyzed matrices, mostly weak correlations were observed between OPs and PYRs insecticides, with the exception of prothiofos in children’s and guardian’s wristbands that showed moderate correlations with deltamethrin in guardian’s (Rs, 0.46) and cypermethrin in children’s wristbands (Rs, −0.43), respectively.

When examining the correlations between urine biomarker levels and the active ingredient levels measured in wristbands, weak correlations were observed for both OPs insecticides (Rs, −0.19 to 0.35) and PYRs insecticides (Rs, −0.12 to 0.08). Chlorpyrifos and diazinon were the only two compounds targeted in urine, wristbands, and dust. Dust levels of chlorpyrifos were moderately correlated with their specific urinary biomarkers TCPy and DETP (Rs, 0.44 and 0.42, respectively) but weakly correlated with the levels measured in wristbands (Rs, 0.16–0.17). In contrast, diazinon dust levels showed no correlation with its specific urinary biomarker IMPy (Rs, −0.02) and were weakly correlated to the wristband levels (Rs, −0.11 to −0.18).

### Spatial clustering of exposure mixtures

**Figure 4.**
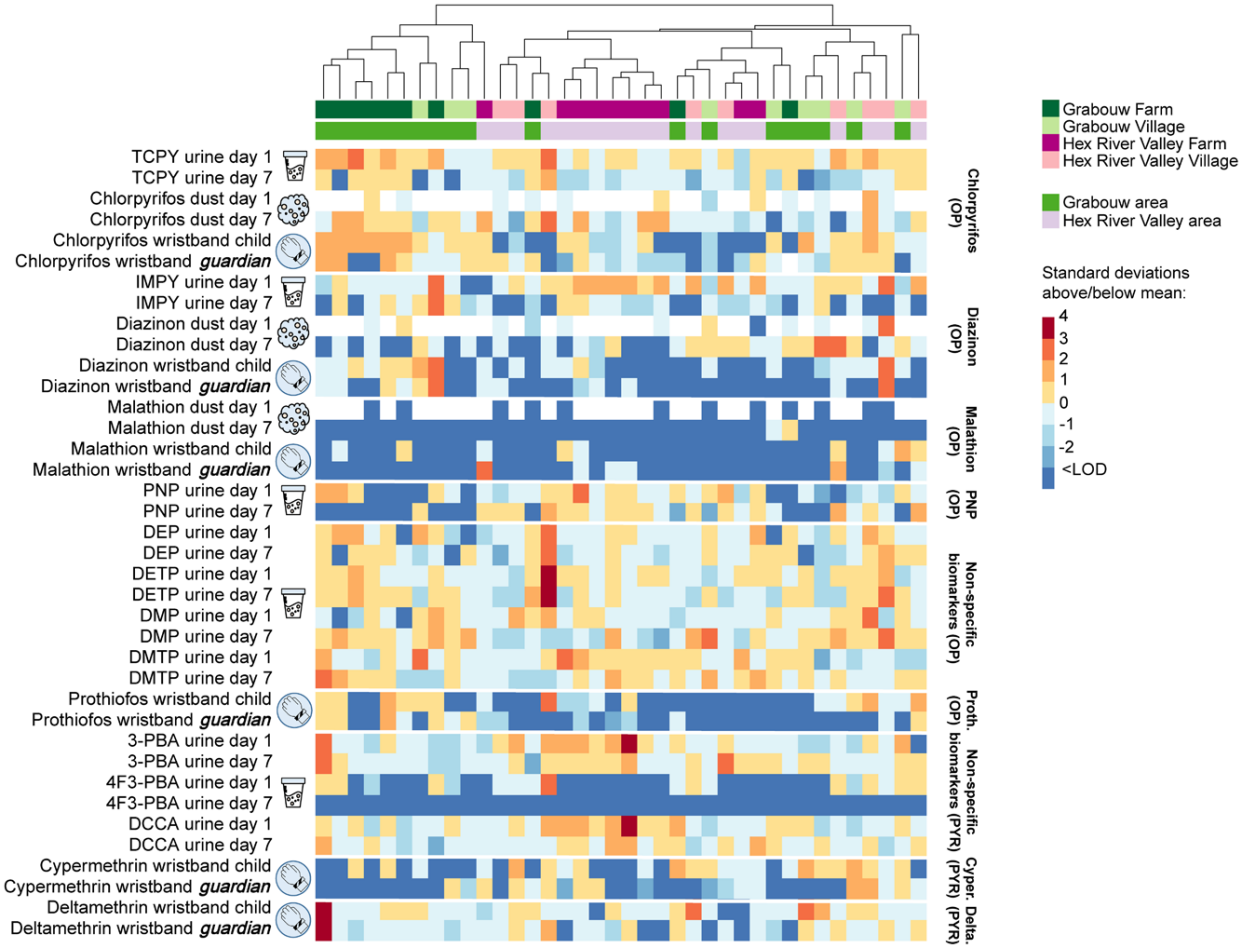
Heatmap showing standardized levels for all insecticides across each urine and dust sample from child and guardian wristbands on day 1 and day 7. Households are ordered based on hierarchical clustering using Spearman’s rank correlation. Blank spaces: households where dust samples were not measured.

Using the hierarchical cluster analysis, we observed that within each study area (i.e., Grabouw or Hex River Valley), the individuals living there tend to cluster based on their similar insecticide mixture profiles across measured matrices (Figure 4). Nevertheless, there are no clear exposure patterns distinguishing individuals living in the two different agricultural areas. A similar result was observed for residents living in either farm or village households. Even though particular clusters of individuals were found within each setting, these differences are not always clear.

The results from the LME models further emphasize this. The overall model (i.e., combining all pesticide levels across matrices) showed no statistical significant difference in exposure levels among residents living in different areas and settings. When looking at individual matrices, statistical differences were only observed for all joint biomarkers’ levels with children living in Hex River Valley, having significantly higher biomarker (log transformed) concentrations than children living in Grabouw (β estimate, 0.36 µg/g creatinine, Table S7; http://links.lww.com/EE/A252). Further spatial differences were observed at the individual pesticide/biomarker level for four urinary biomarkers, one OP in wristbands, and the two OPs measured in dust (Table 3). The log concentrations of PNP and 3-PBA biomarkers were significantly higher among children in Hex River Valley (β estimate, 2.44 and 0.45 µg/g creatinine, respectively). On the other hand, chlorpyrifos log concentrations in children’s wristbands were significantly lower in Hex River Valley than in Grabouw (β estimate, −1.67 ng/g wristband). DMP in urine and diazinon in dust were measured in significantly higher concentrations in villages (β estimate, 0.45 µg/g creatinine and 1.69 ng/g dust, respectively). Still, the opposite was found for DMTP in urine and chlorpyrifos in dust, with village residents showing lower exposure levels than farm residents (β estimate, −0.32 µg/g creatinine and −1.28 ng/g dust, respectively).

**Table 3.** Linear mixed-effect model to study the effect of area and location on the overall log-standardized concentrations and models stratified per matrix and per pesticide within each matrix

	Predictor	β estimate	95% CI	*P* value
Urinary biomarkers (log µg/g creatinine)				
TCPy	Setting^a^: Village	−0.47	−1.40, 0.45	0.33
	Area^b^: Hex River Valley	−0.15	−1.07, 0.78	0.76
	Sex^c^: Male	−0.05	−0.99, 0.89	0.92
IMPy	Setting: Village	−0.42	−1.25, 0.40	0.32
	Area: Hex River Valley	0.36	−0.47, 1.19	0.40
	Sex: Male	0.57	−0.27, 1.41	0.20
PNP	Setting: Village	0.01	−0.74, 0.76	0.98
	Area: Hex River Valley	2.44	1.69, 3.19	<0.001
	Sex: Male	0.31	−0.44, 1.07	0.43
DEP	Setting: Village	−0.11	−0.54, 0.33	0.64
	Area: Hex River Valley	0.09	−0.35, 0.52	0.70
	Sex: Male	−0.18	−0.62, 0.26	0.43
DETP	Setting: Village	−0.20	−0.69, 0.28	0.42
	Area: Hex River Valley	−0.08	−0.57, 0.41	0.75
	Sex: Male	−0.21	−0.71, 0.3	0.41
DMP	Setting: Village	0.45	0.11, 0.79	0.01
	Area: Hex River Valley	−0.24	−0.58, 0.11	0.19
	Sex: Male	0.06	−0.29, 0.40	0.76
DMTP	Setting: Village	−0.32	−0.64, −0.01	0.05
	Area: Hex River Valley	0.06	−0.25, 0.38	0.71
	Sex: Male	−0.13	−0.45, 0.19	0.44
3-PBA	Setting: Village	−0.17	−0.55, 0.21	0.39
	Area: Hex River Valley	0.45	0.07, 0.83	0.03
	Sex: Male	−0.14	−0.52, 0.24	0.48
DCCA	Setting: Village	−0.33	−0.67, 0.01	0.07
	Area: Hex River Valley	0.33	−0.01, 0.67	0.06
	Sex: Male	0.09	−0.26, 0.42	0.63
Wristband pesticides (log ng/g wristband)				
Chlorpyrifos	Setting: Village	0.00	−1.37, 1.37	0.99
	Area: Hex River Valley	−1.67	−3.05, −0.29	0.02
	Sex: Male	0.51	−0.89, 1.91	0.48
Diazinon	Setting: Village	0.13	−1.68, 1.94	0.89
	Area: Hex River Valley	−1.57	−3.39, 0.24	0.10
	Sex: Male	0.08	−1.76, 1.93	0.93
Prothiofos	Setting: Village	0.56	−1.41, 2.54	0.58
	Area: Hex River Valley	−1.29	−3.28, 0.69	0.21
	Sex: Male	0.76	−1.25, 2.77	0.46
Cypermethrin	Setting: Village	0.99	−0.49, 2.48	0.20
	Area: Hex River Valley	0.85	−0.65, 2.34	0.27
	Sex: Male	−0.73	−2.23, 0.78	0.35
Deltamethrin	Setting: Village	0.22	−0.39, 0.82	0.49
	Area: Hex River Valley	−0.59	−1.20, 0.01	0.06
	Sex: Male	−0.26	−0.88, 0.35	0.41
Household dust pesticides (log ng/g dust)				
Chlorpyrifos	Setting: Village	−1.28	−2.30, −0.26	0.02
	Area: Hex River Valley	0.28	−0.74, 1.29	0.59
Diazinon	Setting: Village	1.69	0.74, 2.65	0.001
	Area: Hex River Valley	−0.43	−1.39, 0.52	0.38

The ID was taken as a random effect.

a Farm was taken as the reference value.

b Grabouw was taken as the reference value.

c Female was taken as the reference value.

CI indicates confidence interval.

### Health-risk assessment based on urinary biomonitoring levels

The median (IQR), 95th percentile, and max EDI (µg/kg/d), HQ, and HI (unitless) calculated on days 1 and 7 for each pesticide and pesticide group are presented in Table S5; http://links.lww.com/EE/A252. We observed a high variability in the estimated health risk from single and cumulative pesticide exposure depending on the urine samples used (i.e., samples collected on day 1 vs. day 7). Based on the urine measurements collected on day 1, five participants were estimated to be above the health-risk thresholds (HQ > 1) due to diazinon exposure and two participants due to chlorpyrifos and parathion exposure (one participant each, Figure S2; http://links.lww.com/EE/A252). Also, eight children were above health-risk thresholds due to cumulative exposure to OPs (HI > 1). On the other hand, using the urine measurements collected on day 7, only three participants were above the threshold due to single OPs exposure (two due to diazinon exposure and one due to chlorpyrifos exposure). Similarly, the number of children above the health-risk threshold due to cumulative OPs exposure on day 7 decreased to four participants. Single and cumulative exposure to PYRs was not above risk thresholds in any measured days (Figure S2; http://links.lww.com/EE/A252).

## Discussion

Over a week, we conducted an in-depth exposure assessment using two personal exposure matrices (i.e., biomonitoring [urine samples] and point-of-contact exposure assessment [wristbands]) and one indirect method (household dust) to understand children’s exposure to OPs and PYRs in agricultural areas of South Africa.

Of all the OPs and PYRs analyzed across matrices, 83% were detected at least once, with chlorpyrifos showing the highest concentrations in both wristbands and household dust samples. This is in line with recent research showing a high detection frequency of multiple OPs and PYRs measured in silicone wristbands of children and adolescents living in Latina farm-worker communities in California^38,71^ and North Carolina^67,72,73^ but also in several Peruvian agricultural communities,^74^ suggesting the broad use of these insecticides in agriculture. For both OPs and PYRs, the urinary levels observed in this study were lower than those previously reported for farm workers in the Western Cape region,^9,35^ but the same order of magnitude as those reported for other children living in the same study areas 10 years previous to our study,^21^ likely a result of the similar exposure pathways throughout the years.

A strong temporal agreement between repeated samples was observed for chlorpyrifos in the dust (ICC, 0.92), and moderate temporal correlations were additionally observed for TCPy and two nonspecific OPs and PYRs urinary biomarkers (DETP and 3-PBA, respectively) (Rs, 0.41–0.45). Recent studies conducted in the Western Cape have shown the presence of chlorpyrifos in different environmental matrices (e.g., soil^16,55,75^), emphasizing its ubiquity in the region. Moreover, not only is this pesticide known to be applied on crops,^55^ but it is also known to be used for household pest control.^22,51^ This could lead to an accumulation of household dust particles and continuous exposure of the study population to this insecticide.

Yet, no temporal correlation was found for 69% of the urinary biomarkers analyzed, and only DETP showed a moderate temporal agreement between levels measured 7 days apart. Most of the studied insecticides are very quickly metabolized and excreted from the body after exposure (half-life in the order of hours),^34,76^ which could result in low agreement between repeated measurements. Also, high temporal variability in pesticide exposure is likely to occur even within short time windows (e.g., due to pesticide drift and food exposure), further contributing to these results. Therefore, caution should be taken when using and interpreting single urine measurements as a proxy for long-term exposure.^77^

Only a few studies have compared measured levels of pesticides in different matrices.^28,29,66,78–83^ To the best of our knowledge, this is the first study comparing pesticide levels in urine, wristbands, and household dust. Comparing different exposure matrices enhances the understanding of exposure pathways and how these correlate. Weak correlations were observed between OP and PYR insecticides measured in urine and wristband, suggesting no (linear) relationship between OPs and PYRs exposure. This could be related to intrinsic pesticide characteristics (i.e., different half-lives in soil or molecular weights) that could further influence exposure (e.g., via spray drift) but it could also be related to the application of different pesticide mixtures for different purposes or crops. Additionally, this study is limited in the number of PYRs targeted, which could further lead to underestimating this relationship. For both OPs and PYRs, weak correlations were found between personal exposure matrices (i.e., urine and wristbands), a finding that has also been observed in other studies comparing wristband levels with spot urine samples.^40,84^ This lack of agreement can be partly explained by the different exposure routes (e.g., dietary exposure is not measured using wristbands^41^) or the different exposure-time windows captured by each matrix (i.e., urinary biomarkers are indicators of short-term exposures,^34,76^ whereas wristbands represent cumulative exposures over time). Also, this study was limited by the collection of nonfirst-morning-void spot urine samples, which (although still providing important information on pesticide exposure occurrence) can underestimate significantly accurate estimations on the excreted concentration of pesticides that vary along the day.^85^ Hence, future studies would benefit from multiple urine samples or 24-hour urine collection to bridge the exposure window gap between urine biomarkers and wristbands. Finally, although wristbands have been widely used to measure exposure to several types of chemicals, there is remaining uncertainty regarding the extent to which some external factors (e.g., temperature, humidity, compound properties, sampling rates, or the partition coefficient between silicone and air) may influence the uptake (or release after previous uptake) of pesticides in wristbands.^38,86,87^

Only chlorpyrifos and diazinon were targeted in urine, wristbands, and dust. TCPy showed moderate positive correlations with chlorpyrifos measured in dust, suggesting that home exposure via contaminated dust particles can be a relevant exposure route to chlorpyrifos. In-depth studies would be valuable to understand the actual contribution of dust ingestion to urinary biomarker levels^44,88^ as this agreement might be due to co-occurrence and not necessarily a cause-effect relationship. The dust sample collection was limited to the child’s bedroom floor, which may not represent the entire household.^89^ It is also worth noting that TCPy^66^ and DAP metabolites^29^ have been found in environmental matrices (e.g., dust) as a degradation product of their parent compounds. Therefore, the urinary levels might not only be a result of exposure to chlorpyrifos or other OPs but also to the metabolites themselves. IMPy, on the other hand, did not correlate with diazinon measured in dust and wristbands, suggesting that different exposure routes (e.g., ingestion of contaminated food or water) are likely to play a more prominent role in children’s exposure to diazinon.

We observed that individuals living in the same areas and household settings tend to cluster based on their exposure levels measured across all matrices. Considering the Western Cape’s divided agricultural landscape, with Grabouw being dominated by pome fruits and Hex River Valley producing mainly table grapes,^53^ such clusters were a priori expected.^16,37,55,75^ Different exposure clusters for farm and village residents were also expected^9,21,24^ due to their proximity to agricultural fields treated with pesticides^23,24^ and the fact that most of the farm children in this study reported participating in agricultural activities, potentially enhancing exposure levels.^21,80,90^ However, most of these spatial variances were statistically insignificant, suggesting that the residential location (area and setting) might not be the primary exposure predictor to the insecticides in question and that children residing in different locations share similar exposure patterns. It is worth noting that this study was limited by the narrow sample size, hence the significance of the results should be interpreted with caution. Future studies with larger sample sizes would be beneficial to identify crucial exposure routes and factors.

Finally, we estimated that several children could be at high risk of adverse health effects due to exposure to diazinon, chlorpyrifos, and parathion (HQ > 1) and cumulative exposure to OPs. Although our measured levels vary from day 1 to day 7 considerably, for some individuals, their levels were, at both visits, elevated above the risk thresholds for chlorpyrifos, diazinon, and cumulative exposure to OPs. Moreover, capturing nonfirst-morning-void samples (used in this study) has been shown to underestimate the percentage of children with estimated doses exceeding the recommended guidelines (compared with 24-hour samples).^85^ Hence, the presented risks of OPs are likely to be underestimated in our study. As previously stated, early-life exposure to this chemical group of insecticides has been linked to impaired neurocognitive function and other adverse health effects. It is important to improve policies and create awareness to reduce exposure levels in such risk groups. In contrast, single and cumulative exposure to PYRs were not above risk thresholds. This is aligned with what has been reported in Europe, where human biomonitoring data revealed low health concerns related to PYR exposure.^91^ Nevertheless, it should be noted that in this study, the calculated exposure is likely to be poorly estimated as specific PYR biomarkers (e.g., *cis*-DBCA,^91^ the specific biomarker for deltamethrin) were not available for use. Therefore, further studies tackling this limitation will allow us to understand better the health risks of single and cumulative exposure to PYRs for our population.

In conclusion, this study shows how exposure estimates from different matrices are compared in low- and middle-income settings. We observed that children and their guardians are exposed to OPs and PYRs insecticides, which were measured in urine, wristbands, and household dust samples. Little temporal agreement was observed for most urinary biomarkers, reflecting a high within-individual variability over a 1-week sampling during pesticide spraying season. Therefore, interpreting single urine measurements as exposure proxies to assess the association with chronic health effects has inherent challenges. Low agreements were found between personal exposure matrices (i.e., urinary biomarkers and wristbands), likely due to the different exposure routes targeted by each matrix and the different exposure-time frames captured by these methods. Finally, we showed that during the main spraying season, 21% of our study population was estimated to be above the risk threshold of adverse health effects due to cumulative exposure to OPs. Therefore, efforts are needed to reduce children’s exposure to this group of pesticides. Additional studies are necessary to understand the main risk factors associated with higher exposure levels.

## Conflicts of interest statement

The authors declare that they have no conflicts of interest with regard to the content of this report.

## Acknowledgments

We gratefully acknowledge the study participants and the whole CapSA team, in particular Barblin Michelson, Keith Van Aarde, Neville Peterson, Kharan Vanmali, Wisdom Basera, and Phillancia Januarie, for their active involvement in the data collection. We would also like to thank Andrea Gomez Chamorro and Min Yang for their help with the data analysis and Professor Nicole Probst-Hensch and Professor Roel Vermeulen for their help with the funding acquisition.

## Supplementary Material

## References

[1] Sabzevari S Hofman J. A worldwide review of currently used pesticides’ monitoring in agricultural soils. Sci Total Environ. 2022;812:152344. 34919921 10.1016/j.scitotenv.2021.152344

[2] Rauch S Bradman A Coker E. Determinants of exposure to pyrethroid insecticides in the VHEMBE cohort, South Africa. Environ Sci Technol. 2018;52:12108–12121. 30991471 10.1021/acs.est.8b02767

[3] Murray J Eskenazi B Bornman R. Exposure to DDT and hypertensive disorders of pregnancy among South African women from an indoor residual spraying region: the VHEMBE study. Environ Res. 2018;162:49–54. 29287179 10.1016/j.envres.2017.12.006 PMC6118349

[4] Fuhrimann S Farnham A Staudacher P. Exposure to multiple pesticides and neurobehavioral outcomes among smallholder farmers in Uganda. Environ Int. 2021;152:106477. 33756429 10.1016/j.envint.2021.106477

[5] Hu P Su W Vinturache A. Urinary 3-phenoxybenzoic acid (3-PBA) concentration and pulmonary function in children: a National Health and Nutrition Examination Survey (NHANES) 2007–2012 analysis. Environ Pollut. 2021;270:116178. 33341554 10.1016/j.envpol.2020.116178

[6] Sagiv SK Bruno JL Baker JM. Prenatal exposure to organophosphate pesticides and functional neuroimaging in adolescents living in proximity to pesticide application. Proc Natl Acad Sci USA. 2019;116:18347–18356. 31451641 10.1073/pnas.1903940116 PMC6744848

[7] Shrestha S Parks CG Goldner WS. Incident thyroid disease in female spouses of private pesticide applicators. Environ Int. 2018;118:282–292. 29908479 10.1016/j.envint.2018.05.041 PMC6396853

[8] Suárez B Vela-Soria F Castiello F. Organophosphate pesticide exposure, hormone levels, and interaction with PON1 polymorphisms in male adolescents. Sci Total Environ. 2021;769:144563. 33485193 10.1016/j.scitotenv.2020.144563

[9] Mwanga HH Dalvie MA Singh TS Channa K Jeebhay MF. Relationship between pesticide metabolites, cytokine patterns, and asthma-related outcomes in rural women workers. Int J Environ Res Public Health. 2016;13:957. 27690066 10.3390/ijerph13100957 PMC5086696

[10] Pascale A Laborde A. Impact of pesticide exposure in childhood. Rev Environ Health. 2020;35:221–227. 32598326 10.1515/reveh-2020-0011

[11] Eskenazi B An S Rauch SA. Prenatal exposure to DDT and pyrethroids for malaria control and child neurodevelopment: the VHEMBE cohort, South Africa. Environ Health Perspect. 2018;126:047004-1–047004-11. 29648420 10.1289/EHP2129 PMC6071803

[12] Dalsager L Fage-Larsen B Bilenberg N. Maternal urinary concentrations of pyrethroid and chlorpyrifos metabolites and attention deficit hyperactivity disorder (ADHD) symptoms in 2-4-year-old children from the Odense Child Cohort. Environ Res. 2019;176:108533. 31229776 10.1016/j.envres.2019.108533

[13] Yu CJ Du JC Chiou HC. Increased risk of attention-deficit/hyperactivity disorder associated with exposure to organophosphate pesticide in Taiwanese children. Andrology. 2016;4:695–705. 27070915 10.1111/andr.12183

[14] Von Ehrenstein OS Ling C Cui X. Prenatal and infant exposure to ambient pesticides and autism spectrum disorder in children: population based case-control study. BMJ. 2019;364:l962. 30894343 10.1136/bmj.l962 PMC6425996

[15] Atabila A Phung DT Hogarh JN Sadler R Connell D Chu C. Health risk assessment of dermal exposure to chlorpyrifos among applicators on rice farms in Ghana. Chemosphere. 2018;203:83–89. 29609105 10.1016/j.chemosphere.2018.03.121

[16] Degrendele C Klánová J Prokeš R. Current use pesticides in soil and air from two agricultural sites in South Africa: implications for environmental fate and human exposure. Sci Total Environ. 2021;807:150455. 34634720 10.1016/j.scitotenv.2021.150455

[17] Lisouza FA Owuor PO Lalah JO. Sources, distribution, and risk assessment of organochlorine pesticides in Nairobi City, Kenya. J Environ Sci. 2020;96:178–185. 10.1016/j.jes.2020.04.046 32819692

[18] Akoto O Gavor S Appah MK Apau J. Estimation of human health risk associated with the consumption of pesticide-contaminated vegetables from Kumasi, Ghana. Environ Monit Assess. 2015;187. 10.1007/s10661-015-4471-0 25864079

[19] Chetty-Mhlanga S Fuhrimann S Basera W Eeftens M Röösli M Dalvie MA. Association of activities related to pesticide exposure on headache severity and neurodevelopment of school-children in the rural agricultural farmlands of the Western Cape of South Africa. Environ Int. 2021;146:106237. 33171379 10.1016/j.envint.2020.106237

[20] UNICEF. Understanding the Impacts of Pesticides on Children: A Discussion Paper; 2018.

[21] Molomo RN Basera W Chetty-Mhlanga S. Relation between organophosphate pesticide metabolite concentrations with pesticide exposures, socio-economic factors and lifestyles: a cross-sectional study among school boys in the rural Western Cape, South Africa. Environ Pollut. 2021;275:116660. 33582632 10.1016/j.envpol.2021.116660

[22] Godsmark CN Rother HA. Part of the solution–an engaged scholarship approach for the sustainable reduction of street pesticides and poisonings. Glob Public Health. 2019;14:1535–1545. 30887912 10.1080/17441692.2019.1590619

[23] Figueiredo DM Duyzer J Huss A. Spatio-temporal variation of outdoor and indoor pesticide air concentrations in homes near agricultural fields. Atmos Environ. 2021;262:118612.

[24] Figueiredo DM Nijssen R Krop EJM. Pesticides in doormat and floor dust from homes close to treated fields: spatio-temporal variance and determinants of occurrence and concentrations. Environ Pollut. 2022;301. 10.1016/j.envpol.2022.119024 35202764

[25] Hyland C Laribi O. Review of take-home pesticide exposure pathway in children living in agricultural areas. Environ Res. 2017;156:559–570. 28437652 10.1016/j.envres.2017.04.017

[26] Gaspar FW Chevrier J Quirós-Alcalá L. Levels and determinants of DDT and DDE exposure in the VHEMBE cohort. Environ Health Perspect. 2017;125:1–10. 28696207 10.1289/EHP353 PMC5744723

[27] Becker K Seiwert M Angerer J. GerES IV pilot study: assessment of the exposure of German children to organophosphorus and pyrethroid pesticides. Int J Hyg Environ Health. 2006;209:221–233. 16461005 10.1016/j.ijheh.2005.12.002

[28] Quirós-Alcalá L Bradman A Smith K. Organophosphorous pesticide breakdown products in house dust and children’s urine. J Expo Sci Environ Epidemiol. 2012;22:559–568. 22781438 10.1038/jes.2012.46 PMC4133088

[29] Tamaro CM Smith MN Workman T Griffith WC Thompson B Faustman EM. Characterization of organophosphate pesticides in urine and home environment dust in an agricultural community. Biomarkers. 2018;23:174–187. 29047308 10.1080/1354750X.2017.1395080 PMC6141016

[30] Figueiredo DM Krop EJM Duyzer J. Pesticide exposure of residents living close to agricultural fields in the Netherlands: protocol for an observational study. JMIR Res Protoc. 2021;10:e27883. 33908892 10.2196/27883 PMC8116989

[31] van Wendel de Joode B Barraza D Ruepert C. Indigenous children living nearby plantations with chlorpyrifos-treated bags have elevated 3,5,6-trichloro-2-pyridinol (TCPy) urinary concentrations. Environ Res. 2012;117:17–26. 22749112 10.1016/j.envres.2012.04.006

[32] Rohitrattana J Siriwong W Robson M Panuwet P Barr DB Fiedler N. Pyrethroid insecticide exposure in school-aged children living in rice and aquacultural farming regions of Thailand. Risk Manag Healthc Policy. 2014;7:211–217. 25395873 10.2147/RMHP.S67208 PMC4227621

[33] Barr DB Wang RY Needham LL. Biologic monitoring of exposure to environmental chemicals throughout the life stages: requirements and issues for consideration for the National Children’s Study. Environ Health Perspect. 2005;113:1083–1091. 16079083 10.1289/ehp.7617 PMC1280353

[34] Barr DB. Biomonitoring of exposure to pesticides. J Chem Health Saf. 2008;15:20–29.

[35] Motsoeneng PM Dalvie MA. Relationship between urinary pesticide residue levels and neurotoxic symptoms among women on farms in the Western Cape, South Africa. Int J Environ Res Public Health. 2015;12:6281–6299. 26042367 10.3390/ijerph120606281 PMC4483701

[36] Kapka-Skrzypczak L Cyranka M Skrzypczak M Kruszewski M. Biomonitoring and biomarkers of organophosphate pesticides exposure - state of the art. Ann Agric Environ Med. 2011;18:294–303. 22216802

[37] Fuhrimann S Mol HGJ Dias J. Quantitative assessment of multiple pesticides in silicone wristbands of children/guardian pairs living in agricultural areas in South Africa. Sci Total Environ. 2022;812:152330. 34906574 10.1016/j.scitotenv.2021.152330

[38] Harley KG Parra KL Camacho J. Determinants of pesticide concentrations in silicone wristbands worn by Latina adolescent girls in a California farmworker community: the COSECHA youth participatory action study. Sci Total Environ. 2019;652:1022–1029. 30380470 10.1016/j.scitotenv.2018.10.276 PMC6309742

[39] O’Connell SG Kincl LD Anderson KA. Silicone wristbands as personal passive samplers. Environ Sci Technol. 2014;48:3327–3335. 24548134 10.1021/es405022f PMC3962070

[40] Wacławik M Rodzaj W Wielgomas B. Silicone wristbands in exposure assessment: analytical considerations and comparison with other approaches. Int J Environ Res Public Health. 2022;19:1935. 35206121 10.3390/ijerph19041935 PMC8872583

[41] Wang S Romanak KA Stubbings WA. Silicone wristbands integrate dermal and inhalation exposures to semi-volatile organic compounds (SVOCs). Environ Int. 2019;132:105104. 31465955 10.1016/j.envint.2019.105104 PMC6774250

[42] Hoppin JA Adgate JL Eberhart M Nishioka M Ryan PB. Environmental exposure assessment of pesticides in farmworker homes. Environ Health Perspect. 2006;114:929–935. 16759997 10.1289/ehp.8530 PMC1480520

[43] US EPA. Guidelines for Exposure Assessment. Risk Assessment Forum, Washington, DC. vol. 57; 1992. Available at: http://ngha.med.sa/English/MedicalCities/AlMadinah/MedicalDepartments/Pages/default.aspx.

[44] Degrendele C Prokeš R Šenk P. Human exposure to pesticides in dust from two agricultural sites in South Africa. Toxics. 2022;10:629. 36287909 10.3390/toxics10100629 PMC9610731

[45] Özkaynak H Glen G Cohen J. Model based prediction of age-specific soil and dust ingestion rates for children. J Expo Sci Environ Epidemiol. 2022;32:472–480. 35039613 10.1038/s41370-021-00406-5 PMC9119852

[46] Xue J Zartarian VG Glen G Smith L. Modeled estimates of soil and dust ingestion rates for children. Risk Anal. 2011;31:592–608. 21039709 10.1111/j.1539-6924.2010.01524.x

[47] Yang Y-Q Yiin LM. Daily intake estimation for young children’s ingestion of residential dust and soils contaminated with chlorpyrifos and cypermethrin in Taiwan. Int J Environ Res Public Health. 2018;15:1327–1328. 29941803 10.3390/ijerph15071327 PMC6069238

[48] Waheed S Halsall C Sweetman AJ Jones KC Malik RN. Pesticides contaminated dust exposure, risk diagnosis and exposure markers in occupational and residential settings of Lahore, Pakistan. Environ Toxicol Pharmacol. 2017;56:375–382. 29127912 10.1016/j.etap.2017.11.003

[49] Dabrowski JM. Development of pesticide use maps for South Africa. S Afr J Sci. 2015;111:1–7.

[50] OECD & FAO. OECD-FAO Agricultural Outlook 2016-2025. OECD and FAO; 2016.

[51] Rother H-A. Falling through the regulatory cracks: street selling of pesticides and poisoning among urban youth in South Africa. Int J Occup Environ Health. 2010;16:183–194. 10.1179/107735210799160336 20465065

[52] Western Cape Department of Agriculture. Western Cape Agricultural Sector Profile 2020; 2021.

[53] Chetty-Mhlanga S Basera W Fuhrimann S. A prospective cohort study of school-going children investigating reproductive and neurobehavioral health effects due to environmental pesticide exposure in the Western Cape, South Africa: study protocol. BMC Public Health. 2018;18:1–13. 10.1186/s12889-018-5783-0 PMC6042376 29996806

[54] Fišerová PS Kohoutek J Degrendele C Dalvie MA Klánová J. New sample preparation method to analyse 15 specific and non-specific pesticide metabolites in human urine using LC-MS/MS. J Chromatogr B Analyt Technol Biomed Life Sci. 2021;1166:122542. 10.1016/j.jchromb.2021.122542 33540146

[55] Curchod L Oltramare C Junghans M. Temporal variation of pesticide mixtures in rivers of three agricultural watersheds during a major drought in the Western Cape, South Africa. Water Res X. 2020;6:100039. 31891151 10.1016/j.wroa.2019.100039 PMC6931231

[56] Dalvie MA Africa A London L. Change in the quantity and acute toxicity of pesticides sold in South African crop sectors, 1994–1999. Environ Int. 2009;35:683–687. 19185919 10.1016/j.envint.2008.12.004 PMC2727656

[57] Dalvie MA Africa A Solomons A London L Brouwer D Kromhout H. Pesticide exposure and blood endosulfan levels after first season spray amongst farm workers in the Western Cape, South Africa. J Environ Sci Health B. 2009;44:271–277. 19280480 10.1080/03601230902728351

[58] Tolosana S Rother HA London L. Child’s play: exposure to household pesticide use among children in rural, urban and informal areas of South Africa. S Afr Med J. 2009;99:180–184. 19563096

[59] Akoglu H. User’s guide to correlation coefficients. Turk J Emerg Med. 2018;18:91–93. 30191186 10.1016/j.tjem.2018.08.001 PMC6107969

[60] Koo TK Li MY. A guideline of selecting and reporting intraclass correlation coefficients for reliability research. J Chiropr Med. 2016;15:155–163. 27330520 10.1016/j.jcm.2016.02.012 PMC4913118

[61] Atabila A Sadler R Phung DT. Biomonitoring of chlorpyrifos exposure and health risk assessment among applicators on rice farms in Ghana. Environ Sci Pollut Res Int. 2018;25:20854–20867. 29766419 10.1007/s11356-018-2259-9

[62] Fernández S F Pardo O Corpas-Burgos F Yusà V. Exposure and cumulative risk assessment to non-persistent pesticides in Spanish children using biomonitoring. Sci Total Environ. 2020;746:140983. 32750575 10.1016/j.scitotenv.2020.140983

[63] van Wendel de Joode B Mora AM Córdoba L. Aerial application of mancozeb and urinary ethylene thiourea (ETU) concentrations among pregnant women in Costa Rica: the Infants’ Environmental Health Study (ISA). Environ Health Perspect. 2014;122:1321–1328. 25198283 10.1289/ehp.1307679 PMC4256696

[64] Beckford K Grimes CA Margerison C. A systematic review and meta-analysis of 24-h urinary output of children and adolescents: impact on the assessment of iodine status using urinary biomarkers. Eur J Nutr. 2020;59:3113–3131. 31784814 10.1007/s00394-019-02151-w PMC7501103

[65] Yusa V Millet M Coscolla C Pardo O Roca M. Occurrence of biomarkers of pesticide exposure in non-invasive human specimens. Chemosphere. 2015;139:91–108. 26070147 10.1016/j.chemosphere.2015.05.082

[66] Morgan MK Sheldon LS Croghan CW. Exposures of preschool children to chlorpyrifos and its degradation product 3,5,6-trichloro-2-pyridinol in their everyday environments. J Expo Anal Environ Epidemiol. 2005;15:297–309. 15367928 10.1038/sj.jea.7500406

[67] European Food Safety Authority (EFSA). Conclusion on the peer review of the pesticide human health risk assessment of the active substance chlorpyrifos. EFSA J. 2014;12:1–34. 10.2903/j.efsa.2014.3485 PMC13159934 42125125

[68] European Food Safety Authority (EFSA). EU Pesticides Database. Available at: https://food.ec.europa.eu/plants/pesticides/eu-pesticides-database_en. Accessed 13 April 2023.

[69] Crivellente F Hart A Hernandez-Jerez AF; European Food Safety Authority (EFSA). Establishment of cumulative assessment groups of pesticides for their effects on the nervous system. EFSA J. 2019;17:e05800. 32626428 10.2903/j.efsa.2019.5800 PMC7009249

[70] Lubin JH Colt JS Camann D. Epidemiologic evaluation of measurement data in the presence of detection limits. Environ Health Perspect. 2004;112:1691–1696. 15579415 10.1289/ehp.7199 PMC1253661

[71] Arcury TA Chen H Quandt SA. Pesticide exposure among Latinx children: comparison of children in rural, farmworker and urban, non-farmworker communities. Sci Total Environ. 2021;763:144233. 33385842 10.1016/j.scitotenv.2020.144233 PMC7855950

[72] Arcury TA Chen H Arnold TJ. Pesticide exposure among Latinx child farmworkers in North Carolina. Am J Ind Med. 2021;64:602–619. 34036619 10.1002/ajim.23258 PMC8819502

[73] Arcury TA Chen H Quandt SA. Pesticide exposure among Latinx children in rural farmworker and urban non-farmworker communities: associations with locality and season. Int J Environ Res Public Health. 2023;20:5647. 10.3390/ijerph20095647 PMC10178580 37174167

[74] Bergmann AJ North PE Vasquez L Bello H Del Carmen Gastañaga Ruiz M Anderson KA. Multi-class chemical exposure in rural Peru using silicone wristbands. J Expo Sci Environ Epidemiol. 2017;27:560–568. 28745304 10.1038/jes.2017.12 PMC5658680

[75] Veludo AF Figueiredo D Degrendele C. Seasonal variations in air concentrations of 27 organochlorine pesticides (OCPs) and 25 current-use pesticides (CUPs) across three agricultural areas of South Africa. Chemosphere. 2021;289:133162. 34875296 10.1016/j.chemosphere.2021.133162

[76] Bravo N Grimalt JO Mazej D Tratnik JS Sarigiannis DA Horvat M. Mother/child organophosphate and pyrethroid distributions. Environ Int. 2020;134:105264. 31706197 10.1016/j.envint.2019.105264

[77] Burns CJ LaKind JS. Elements to increase translation in pyrethroid epidemiology research: a review. Sci Total Environ. 2022;813:152568. 34954171 10.1016/j.scitotenv.2021.152568

[78] Trunnelle KJ Bennett DH Tulve NS. Urinary pyrethroid and chlorpyrifos metabolite concentrations in Northern California families and their relationship to indoor residential insecticide levels, part of the Study of Use of Products and Exposure Related Behavior (SUPERB). Environ Sci Technol. 2014;48:1931–1939. 24422434 10.1021/es403661a

[79] Wilson NK Strauss WJ Iroz-Elardo N Chuang JC. Exposures of preschool children to chlorpyrifos, diazinon, pentachlorophenol, and 2,4-dichlorophenoxyacetic acid over 3 years from 2003 to 2005: a longitudinal model. J Expo Sci Environ Epidemiol. 2010;20:546–558. 19724304 10.1038/jes.2009.45

[80] Glorennec P Serrano T Fravallo M. Determinants of children’s exposure to pyrethroid insecticides in western France. Environ Int. 2017;104:76–82. 28453973 10.1016/j.envint.2017.04.007

[81] Morgan MK Sheldon LS Croghan CW Jones PA Chuang JC Wilson NK. An observational study of 127 preschool children at their homes and daycare centers in Ohio: environmental pathways to *cis*- and *trans*-permethrin exposure. Environ Res. 2007;104:266–274. 17258193 10.1016/j.envres.2006.11.011

[82] Wilson NK Chuang JC Lyu C Menton R Morgan MK. Aggregate exposures of nine preschool children to persistent organic pollutants at day care and at home. J Expo Anal Environ Epidemiol. 2003;13:187–202. 12743613 10.1038/sj.jea.7500270

[83] Tulve NS Egeghy PP Fortmann RC. Multimedia measurements and activity patterns in an observational pilot study of nine young children. J Expo Sci Environ Epidemiol. 2008;18:31–44. 17851450 10.1038/sj.jes.7500600

[84] Samon SM Hammel SC Stapleton HM Anderson KA. Silicone wristbands as personal passive sampling devices: current knowledge, recommendations for use, and future directions. Environ Int. 2022;169:107339. 36116363 10.1016/j.envint.2022.107339 PMC9713950

[85] Hyland C Kogut K Gunier RB. Organophosphate pesticide dose estimation from spot and 24-hr urine samples collected from children in an agricultural community. Environ Int. 2021;146:106226. 33152651 10.1016/j.envint.2020.106226 PMC8168949

[86] Anderson KA Points GL Donald CE. Preparation and performance features of wristband samplers and considerations for chemical exposure assessment. J Expo Sci Environ Epidemiol. 2017;27:551–559. 28745305 10.1038/jes.2017.9 PMC5658681

[87] Donald CE Scott RP Blaustein KL. Silicone wristbands detect individuals’ pesticide exposures in West Africa. R Soc Open Sci. 2016;3:160433. 27853621 10.1098/rsos.160433 PMC5108971

[88] Vanacker M Quindroit P Angeli K. Aggregate and cumulative chronic risk assessment for pyrethroids in the French adult population. Food Chem Toxicol. 2020;143:111519. 32619558 10.1016/j.fct.2020.111519

[89] Jílková S Melymuk L Vojta S Vykoukalová M Bohlin-Nizzetto P Klánová J. Small-scale spatial variability of flame retardants in indoor dust and implications for dust sampling. Chemosphere. 2018;206:132–141. 29734094 10.1016/j.chemosphere.2018.04.146

[90] Thompson B Griffith WC Barr DB Coronado GD Vigoren EM Faustman EM. Variability in the take-home pathway: farmworkers and non-farmworkers and their children. J Expo Sci Environ Epidemiol. 2014;24:522–531. 24594649 10.1038/jes.2014.12 PMC4141015

[91] Tarazona JV Cattaneo I Niemann L. A tiered approach for assessing individual and combined risk of pyrethroids using human biomonitoring data. Toxics. 2022;10:451. 36006130 10.3390/toxics10080451 PMC9416723

