# Supplementary material for: Child exposure to organophosphate and pyrethroid insecticides measured in urine, wristbands, and household dust and its implications for child health in South Africa: A panel study
Source: Environ Epidemiol. 2023 Dec 29;8(1):e282. doi: 10.1097/EE9.0000000000000282 (PMC10852399; doi:10.1097/EE9.0000000000000282)
Supplement: Supplementary file 1 [file ee9-8-e282-s001.docx]

**Supplementary Material**

***Child exposure to*** ***organophosphate and pyrethroid insecticides measured in urine, wristbands and household dust and its implications for child health in South Africa – A panel study***

Adriana Fernandes Veludo^1, 2^, Martin Röösli^1, 2^, Mohamed Aqiel Dalvie^3^, Petra Stuchlík Fišerová^4^, Roman Prokeš^4, 5^, Petra Přibylová^4^, Petr Šenk^4^, Jiří Kohoutek^4^, Mufaro Mugari^3^, Jana Klánová^4^, Anke Huss^6^, Daniel Martins Figueiredo^6^, Hans Mol^7^, Jonatan Dias^7^, Céline Degrendele^4, 8^, Samuel Fuhrimann^1, 2 *^

^1^ Swiss Tropical and Public Health Institute (Swiss TPH), 4123 Allschwil, Switzerland

^2^ University of Basel, 4002, Basel, Switzerland

^3^ Centre for Environmental and Occupational Health Research, School of Public Health and Family Medicine, University of Cape Town, 7925 Cape Town, South Africa

^4^ RECETOX, Faculty of Science, Masaryk University, Kotlarska 2, 611 37 Brno, Czech Republic

^5^ Global Change Research Institute of the Czech Academy of Sciences, 60300 Brno, Czech Republic

^6^ Institute for Risk Assessment Sciences, Utrecht University, Utrecht, the Netherlands

^7^ Wageningen Food Safety Research, part of Wageningen University & Research, Akkermaalsbos 2, 6708 WB, Wageningen, The Netherlands

^8^ Aix-Marseille University, CNRS, LCE, 13003 Marseille, France

*** Corresponding author**: Samuel Fuhrimann, Email: [samuel.fuhrimann@swisstph.ch](mailto:samuel.fuhrimann@swisstph.ch)

**Table of Contents**

[Glossary of urinary metabolites analyzed 3](#_Toc150863233)

[Table S1. Concentrations of OPs and PYRs urinary biomarkers (ng/mL) unadjusted for creatinine, creatinine concentrations (mg/L) and Limit of Detection/Quantification. 4](#_Toc150863234)

[Table S2. OPs and PYRs concentrations in wristbands (ng/g wristband). 6](#_Toc150863235)

[Table S3. OPs concentrations in household dust (ng/g). 8](#_Toc150863236)

[Table S4. Correlation matrix showing the Spearman rank correlations (R*s* upper diagonal, non-italic) and respective *p*-values (lower diagonal, italic) between the levels of organophosphates (OPs) and pyrethroids (PYRs) measured in urine, wristbands and household dust measured at day 1 and day 7. 10](#_Toc150863237)

[Table S5. Parameters used to calculate the estimated daily intake (EDI) using the urine biomonitoring levels. 17](#_Toc150863238)

[Table S6. Descriptive statistics of EDI (µg/kg/day), HQ and HI (unitless) calculated for each pesticide and pesticide group at Day 1 and Day 7. 18](#_Toc150863239)

[Table S7. Linear mixed effect models to study the effect of area and location on the overall log standardized concentrations and models stratified per matrix. The ID was taken as a random effect 19](#_Toc150863240)

[Figure S1. Correlation matrix showing the Spearman rank correlations (R*s*) between the levels of organophosphates (OPs) and pyrethroids (PYRs) in urine, wristbands and dust measured at day 1 and day 7. For *p*-values consult Table S6. 20](#_Toc150863241)

[Figure S2. Estimated Hazard Quotient (A) and Hazard Index (B) for each pesticide and pesticide group, respectively, stratified per day. A value above one (range from light orange to red, i.e., from lower to higher values) indicates a possible risk of health effects due to exposure to a specific pesticide (A) or pesticide group (B). 21](#_Toc150863242)

[References 22](#_Toc150863243)

# **Glossary of urinary metabolites analyzed**

#

| *3-PBA* | 3-phenoxybenzoic acid |
| --- | --- |
| *4F3-PBA* | 4-fluoro-3-phenoxybenzoic acid |
| *CMHC* | 3-chloro-4-methylumbelliferone |
| *DCCA* | cis/trans-3-(2,2-dichlorovinyl)-2,2-dimethyl cyclopropane-carboxylic acid |
| *DEP* | Diethylphosphate |
| *DETP* | Diethylthiophosphate |
| *DEDTP* | Diethyldithiophosphate |
| *DMP* | Dimethylphosphate |
| *DMTP* | Dimethylthiophosphate |
| *IMPy* | 2-isopropyl-4-methyl-6-hydroxypyrimidine |
| *TCPy* | 3,5,6-trichloro-2-pyridinol |
| *PNP* | *p*-nitrophenol |
| *MDA* | Malathion dicarboxylic acid |

# Table S1. Concentrations of OPs and PYRs urinary biomarkers (ng/mL) unadjusted for creatinine, creatinine concentrations (mg/L) and Limit of Detection/Quantification.

|  | **Creatinine** | **3-PBA** | **4F3-PBA** | **CMHC** | **DEDTP** | **MDA** | **PNP** | **t/c-DCCA** | **TCPY** | **DEET** | **IMPY** | **DEP** | **DETP** | **DMP** | **DMTP** |
| --- | --- | --- | --- | --- | --- | --- | --- | --- | --- | --- | --- | --- | --- | --- | --- |
|  | (mg/L) | (ng/mL) | (ng/mL) | (ng/mL) | (ng/mL) | (ng/mL) | (ng/mL) | (ng/mL) | (ng/mL) | (ng/mL) | (ng/mL) | (ng/mL) | (ng/mL) | (ng/mL) | (ng/mL) |
| Day 1 | 1493.9 | 4.45 | <LOD | <LOD | <LOD | <LOD | 5.18 | 4.64 | 1.58 | <LOD | 8.41 | 2.09 | 1.40 | 24.72 | 7.38 |
|  | 524.9 | 1.36 | <LOD | <LOD | <LOD | <LOD | 0.20 | 1.22 | 0.19 | <LOD | 0.49 | <LOD | 0.56 | 5.97 | 6.02 |
|  | 918.9 | 0.82 | <LOD | <LOD | <LOD | <LOD | 0.08 | 0.95 | 0.63 | <LOD | 2.46 | 1.48 | 0.74 | 7.83 | 4.12 |
|  | 851.9 | 1.32 | <LOD | <LOD | <LOD | <LOD | 0.15 | 0.88 | 0.45 | <LOD | 0.93 | 1.89 | 0.75 | 6.05 | 2.19 |
|  | 1963.9 | 0.82 | <LOD | <LOD | <LOD | <LOD | 0.24 | 1.45 | 0.80 | <LOD | <LOD | 2.27 | 0.82 | 21.12 | 3.09 |
|  | 900.9 | 0.80 | <LOD | <LOD | <LOD | <LOD | 0.07 | 0.68 | 0.04 | <LOD | 0.22 | 1.92 | 0.91 | 8.99 | 4.43 |
|  | 750.9 | 1.30 | <LOD | <LOD | <LOD | <LOD | 0.10 | 1.04 | 0.33 | <LOD | 1.83 | <LOD | 0.60 | 12.42 | 2.09 |
|  | 248.9 | 2.38 | <LOD | <LOD | <LOD | <LOD | 0.14 | 3.22 | 0.27 | <LOD | 0.54 | <LOD | 0.19 | 2.45 | 1.07 |
|  | 452.9 | 1.03 | <LOD | <LOD | <LOD | <LOD | 0.23 | 1.03 | 0.88 | <LOD | 1.89 | <LOD | 0.51 | 4.03 | 1.15 |
|  | 930.9 | 0.59 | <LOD | <LOD | <LOD | <LOD | 0.32 | 0.57 | 1.65 | <LOD | 2.45 | 6.37 | 1.02 | 9.33 | 2.19 |
|  | 585.9 | 0.94 | <LOD | <LOD | <LOD | <LOD | 0.57 | 0.44 | 0.79 | <LOD | 2.23 | <LOD | 0.37 | 6.36 | 1.25 |
|  | 1373.9 | <LOD | 1.97 | <LOD | <LOD | <LOD | 0.12 | 0.88 | 3.78 | <LOD | 3.16 | 2.90 | 0.75 | 12.42 | 1.50 |
|  | 966.9 | 0.78 | 0.99 | <LOD | <LOD | <LOD | 0.07 | 0.45 | 3.01 | <LOD | 9.55 | 3.10 | 2.45 | 3.64 | 1.62 |
|  | 613.9 | 0.64 | 0.72 | <LOD | <LOD | <LOD | 0.05 | 0.40 | 6.75 | <LOD | 0.21 | 5.11 | 0.61 | 38.82 | 1.08 |
|  | 770.9 | 2.91 | 3.10 | <LOD | <LOD | <LOD | 0.72 | 1.91 | 87.05 | <LOD | 1.35 | 17.60 | 11.60 | 25.32 | 3.68 |
|  | 993.9 | 2.52 | 1.03 | <LOD | <LOD | <LOD | 0.16 | 1.09 | 1.39 | <LOD | 1.18 | 2.42 | 0.26 | 35.52 | 1.67 |
|  | 774.9 | 0.72 | 1.27 | <LOD | <LOD | <LOD | 0.11 | 0.58 | 1.95 | <LOD | 2.28 | 1.69 | 0.55 | 8.32 | 1.99 |
|  | 826.9 | 0.85 | 0.83 | <LOD | <LOD | <LOD | 0.05 | 0.64 | 2.30 | <LOD | 0.40 | 1.66 | 0.50 | 9.43 | 1.80 |
|  | 606.9 | 0.60 | 0.71 | <LOD | <LOD | <LOD | <LOD | 0.35 | 3.03 | <LOD | 0.40 | 3.08 | 0.64 | 11.32 | 0.84 |
|  | 145.9 | 0.10 | 0.18 | <LOD | <LOD | <LOD | 0.33 | 0.09 | 1.17 | <LOD | 0.09 | <LOD | 0.21 | <LOD | 0.26 |
|  | 819.9 | 1.32 | 0.89 | <LOD | <LOD | <LOD | 0.54 | 2.01 | 3.23 | <LOD | 0.14 | <LOD | 0.54 | 3.35 | 2.63 |
|  | 1323.9 | 0.37 | 0.81 | <LOD | <LOD | <LOD | <LOD | 0.40 | 3.66 | <LOD | 0.27 | 3.47 | 1.99 | 19.42 | 1.04 |
|  | 181.9 | 0.12 | <LOD | <LOD | <LOD | <LOD | <LOD | 0.04 | 0.71 | <LOD | 0.06 | <LOD | <LOD | <LOD | 0.25 |
|  | 256.9 | 1.21 | 0.38 | <LOD | <LOD | <LOD | 0.60 | 0.26 | 4.45 | <LOD | 0.01 | <LOD | 0.40 | <LOD | 2.06 |
|  | 1543.9 | 0.51 | 1.75 | <LOD | <LOD | <LOD | 0.51 | 1.07 | 7.31 | 0.11 | 33.20 | 8.83 | 2.21 | 47.02 | 2.85 |
|  | 322.9 | 0.51 | <LOD | <LOD | <LOD | <LOD | <LOD | 0.49 | 1.74 | <LOD | 0.13 | <LOD | 0.16 | <LOD | 1.02 |
|  | 491.9 | 0.36 | <LOD | <LOD | <LOD | <LOD | 0.23 | 0.44 | 13.03 | <LOD | 0.20 | 4.20 | 1.27 | 2.71 | 0.71 |
|  | 1063.9 | 0.91 | 1.15 | <LOD | <LOD | <LOD | 0.12 | 0.83 | 2.71 | <LOD | 0.16 | 4.63 | 0.88 | 17.02 | 1.51 |
|  | 938.9 | 0.91 | <LOD | <LOD | <LOD | <LOD | <LOD | 1.01 | 1.76 | <LOD | 0.23 | 2.74 | 0.57 | 7.99 | 2.25 |
|  | 1253.9 | 1.26 | 1.36 | <LOD | <LOD | <LOD | 0.43 | 0.44 | 0.73 | <LOD | 0.09 | 5.77 | 0.33 | 9.46 | 2.21 |
|  | 181.9 | 0.10 | <LOD | <LOD | <LOD | <LOD | <LOD | 0.12 | 0.07 | <LOD | <LOD | <LOD | <LOD | <LOD | 0.27 |
|  | 2343.9 | 1.06 | 1.19 | <LOD | <LOD | <LOD | 0.39 | 2.00 | 1.05 | <LOD | 0.84 | 1.72 | 0.91 | 60.92 | 6.70 |
|  | 904.9 | 2.42 | 1.48 | <LOD | <LOD | <LOD | 0.45 | 1.16 | 0.90 | <0,063 | 0.11 | <LOD | 0.31 | 25.62 | 0.96 |
|  | 2893.9 | 0.72 | 1.33 | <LOD | <LOD | <LOD | 0.16 | 0.95 | 4.79 | <LOD | 3.92 | 7.12 | 3.41 | 45.72 | 2.17 |
|  | 1723.9 | 0.75 | <LOD | <LOD | <LOD | <LOD | 0.02 | 0.58 | 1.28 | <LOD | 0.94 | <LOD | 0.48 | 20.32 | 0.59 |
|  | 189.9 | 0.14 | <LOD | <LOD | <LOD | <LOD | <LOD | 0.10 | 0.41 | <LOD | 0.06 | <LOD | 0.13 | 4.99 | 3.64 |
|  | 663.9 | 0.32 | <LOD | <LOD | <LOD | <LOD | 0.06 | 0.81 | 0.67 | <LOD | 0.10 | <LOD | 0.77 | 4.57 | 2.86 |
|  | 154.9 | 0.15 | <LOD | <LOD | <LOD | <LOD | <LOD | 0.15 | 0.58 | <LOD | 0.04 | <LOD | 0.13 | <LOD | 0.50 |
| Day 7 | 577.9 | 0.63 | <LOD | <LOD | <LOD | <LOD | 0.39 | 0.79 | 0.08 | <LOD | 0.33 | <LOD | 0.59 | 4.63 | 1.70 |
|  | 1303.9 | 1.91 | <LOD | 0.06 | <LOD | <LOD | 0.83 | 1.14 | 0.15 | <LOD | 1.42 | <LOD | 0.28 | 7.28 | 2.06 |
|  | 1533.9 | 1.27 | <LOD | <LOD | <LOD | <LOD | 0.33 | 1.37 | 0.27 | <LOD | 0.39 | 1.54 | 0.60 | 4.67 | 2.37 |
|  | 1453.9 | 1.39 | <LOD | <LOD | <LOD | <LOD | 0.45 | 0.89 | 0.28 | <LOD | 0.19 | 2.43 | 0.72 | 5.93 | 5.26 |
|  | 2283.9 | 1.38 | <LOD | <LOD | <LOD | <LOD | 0.99 | 1.30 | 0.78 | 1.75 | 1.12 | 4.43 | 1.25 | 23.15 | 3.79 |
|  | 1203.9 | 1.38 | <LOD | <LOD | <LOD | <LOD | 0.25 | 0.67 | 0.04 | <LOD | 0.09 | 2.04 | 0.67 | 6.57 | 4.97 |
|  | 925.9 | 1.23 | <LOD | <LOD | <LOD | <LOD | 0.24 | 0.56 | 0.28 | <LOD | 0.15 | 1.49 | 0.44 | 5.19 | 2.09 |
|  | 1173.9 | 2.61 | <LOD | <LOD | <LOD | <LOD | 0.51 | 2.63 | 0.42 | <LOD | 0.11 | 2.00 | 0.47 | 11.35 | 3.34 |
|  | 769.9 | 1.03 | <LOD | <LOD | <LOD | <LOD | 0.38 | 2.33 | 0.07 | <LOD | 0.26 | 3.08 | 1.15 | 39.75 | 4.19 |
|  | 1203.9 | 2.32 | <LOD | <LOD | <LOD | <LOD | 1.04 | 2.03 | 1.85 | <LOD | 0.30 | 3.90 | 0.85 | 19.45 | 6.14 |
|  | 1783.9 | 8.50 | <LOD | <LOD | <LOD | <LOD | 0.86 | 1.62 | 0.81 | <LOD | 0.15 | 3.13 | 0.60 | 16.45 | 2.36 |
|  | 1043.9 | 2.13 | <LOD | <LOD | <LOD | <LOD | 1.17 | 1.54 | 1.05 | <LOD | <LOD | 3.39 | 0.49 | 14.95 | 2.74 |
|  | 3813.9 | 1.99 | <LOD | <LOD | <LOD | <LOD | 0.99 | 1.35 | 0.79 | <LOD | <LOD | 25.10 | 20.10 | 260.75 | 8.46 |
|  | 1163.9 | 1.14 | <LOD | <LOD | <LOD | <LOD | 1.02 | 0.95 | 0.57 | <0,063 | <LOD | 5.60 | 1.05 | 22.05 | 1.48 |
|  | 2783.9 | 3.59 | <LOD | <LOD | <LOD | <LOD | 2.86 | 1.68 | 18.91 | <LOD | <LOD | 63.00 | 42.00 | 16.55 | 2.53 |
|  | 4133.9 | 4.98 | <LOD | 0.18 | <LOD | <LOD | 2.37 | 1.91 | 0.45 | <LOD | <LOD | 8.24 | 0.95 | 15.75 | 3.23 |
|  | 5483.9 | 6.35 | <LOD | <LOD | <LOD | <LOD | 2.82 | 1.42 | 0.04 | <LOD | <LOD | 10.40 | 1.89 | 191.75 | 11.50 |
|  | 2893.9 | 1.56 | <LOD | <LOD | <LOD | <LOD | 2.51 | 1.51 | 1.20 | <LOD | <LOD | 1.81 | 0.89 | 10.15 | 5.29 |
|  | 1263.9 | 1.20 | <LOD | <LOD | <LOD | <LOD | 1.28 | 0.62 | 0.16 | <LOD | <LOD | <LOD | 0.39 | 60.05 | 5.74 |
|  | 73.5 | 0.09 | <LOD | <LOD | <LOD | <LOD | <LOD | 0.06 | <LOD | <LOD | 0.06 | <LOD | 0.13 | 2.88 | 0.44 |
|  | 1073.9 | 0.62 | <LOD | <LOD | <LOD | <LOD | <0,015 | 0.99 | 0.34 | <LOD | <LOD | 2.21 | 0.77 | 14.45 | 3.29 |
|  | 262.9 | 0.20 | <LOD | <LOD | <LOD | <LOD | <LOD | 0.24 | 0.34 | <LOD | 0.10 | <LOD | 0.22 | 5.07 | 0.98 |
|  | 1123.9 | 0.88 | <LOD | <LOD | <LOD | <LOD | <LOD | 0.32 | 1.65 | <LOD | 0.48 | 1.80 | 1.07 | 15.05 | 1.01 |
|  | 431.9 | 2.21 | <LOD | <LOD | <LOD | <LOD | <LOD | 1.22 | 1.55 | <LOD | <LOD | <LOD | 0.70 | 12.55 | 7.40 |
|  | 640.9 | 0.20 | <LOD | <LOD | <LOD | <LOD | <LOD | 0.12 | 0.38 | <LOD | 10.10 | 1.49 | 0.86 | 25.65 | 0.67 |
|  | 260.9 | 0.18 | <LOD | <LOD | <LOD | <LOD | <LOD | 0.12 | 0.29 | <LOD | 0.18 | <LOD | 0.22 | 3.59 | 0.65 |
|  | 622.9 | 0.51 | <LOD | <LOD | <LOD | <LOD | <LOD | 0.39 | 1.46 | <LOD | <LOD | 3.10 | 1.51 | 14.55 | 1.90 |
|  | 415.9 | 0.10 | <LOD | <LOD | <LOD | <LOD | <LOD | 0.25 | 1.28 | <LOD | 0.04 | 2.45 | 0.80 | 5.69 | 0.46 |
|  | 455.9 | 0.46 | <LOD | <LOD | <LOD | <LOD | <LOD | 0.25 | 0.45 | <LOD | <LOD | <LOD | 0.49 | 5.31 | 1.01 |
|  | 993.9 | 0.87 | <LOD | <LOD | <LOD | <LOD | <0,015 | 0.62 | 0.49 | <LOD | 0.23 | 3.08 | 0.98 | 111.75 | 6.29 |
|  | 259.9 | 0.14 | <LOD | <LOD | <LOD | <LOD | <LOD | 0.18 | 0.06 | <LOD | 0.03 | <LOD | 0.16 | 9.95 | 0.32 |
|  | 88.9 | 0.04 | <LOD | <LOD | <LOD | <LOD | <LOD | <LOD | <LOD | <LOD | 0.06 | <LOD | 0.15 | <LOD | 0.30 |
|  | 396.9 | 0.41 | <LOD | <LOD | <LOD | <LOD | <LOD | 0.24 | 0.41 | <LOD | 0.20 | <LOD | 0.63 | 6.65 | 1.77 |
|  | 1523.9 | 1.22 | <LOD | <LOD | <LOD | <LOD | 0.31 | 1.18 | 0.16 | <LOD | 0.31 | 3.15 | 1.61 | 26.65 | 2.20 |
|  | 1153.9 | 1.25 | <LOD | <LOD | <LOD | <LOD | <LOD | 1.40 | 0.02 | <LOD | 0.70 | <LOD | 0.20 | 20.45 | 2.40 |
|  | 2123.9 | 1.17 | <LOD | <LOD | <LOD | <LOD | 1.33 | 1.41 | <LOD | <LOD | 3.84 | 2.42 | 0.68 | 31.85 | 1.65 |
|  | 244.9 | 0.29 | <LOD | <LOD | <LOD | <LOD | <LOD | 0.19 | <LOD | <LOD | 0.05 | <LOD | 0.23 | 5.65 | 0.77 |
|  | 1563.9 | 2.30 | <LOD | <LOD | <LOD | <LOD | 0.14 | 1.70 | 0.08 | <LOD | 0.21 | 8.64 | 0.28 | 18.05 | 4.08 |
| **LOD** |  | 0.01 | 0.01 | 0.01 | 0.03 | 0.05 | 0.01 | 0.01 | 0.01 | 0.02 | 0.003 | 0.44 | 0.03 | 0.66 | 0.05 |
| **LOQ** |  | 0.02 | 0.04 | 0.02 | 0.08 | 0.15 | 0.02 | 0.04 | 0.02 | 0.06 | 0.01 | 1.48 | 0.10 | 2.20 | 0.16 |

LOD – Limit of detection; LOQ – Limit of quantification

# Table S2. OPs and PYRs concentrations in wristbands (ng/g wristband).

| **Participant** | **Chlorpyrifos** | **Cypermethrin** | **Deltamethrin** | **Diazinon** | **Malathion** | **Prothiofos** | **Location** | **Area** |
| --- | --- | --- | --- | --- | --- | --- | --- | --- |
| parent | 77.04 | 25.69 | 1.65 | 0.99 | 1.26 | 3.10 | Farm |  |
| parent | 2.71 | 1.54 | <LOD | <LOD | <LOD | <LOD | Farm |  |
| parent | 8.91 | <LOD | <LOD | <LOD | <LOD | <LOD | Farm |  |
| parent | 14.45 | 2.21 | <LOD | <LOD | <LOD | <LOD | Farm |  |
| parent | 167.46 | 25.18 | 3.24 | 4.15 | 14.27 | 4.59 | Farm |  |
| parent | 10.60 | 6.82 | <LOD | <LOD | <LOD | <LOD | Farm |  |
| parent | 48.13 | 35.28 | 2.66 | 2.03 | <LOD | <LOD | Farm |  |
| parent | 25.96 | <LOD | 4.90 | 1.93 | 1.86 | 3.90 | Farm |  |
| parent | 12.71 | <LOD | 1.24 | <LOD | 2.34 | 1.78 | Farm |  |
| parent | 181.08 | 11.36 | 3.45 | <LOD | <LOD | <LOD | Farm |  |
| parent | <LOD | 5.59 | 1.64 | <LOD | <LOD | <LOD | Village |  |
| parent | 26.68 | 7.24 | 5.33 | <LOD | 1.32 | 78.92 | Village |  |
| parent | 64.27 | 9.45 | 8.48 | 75.66 | 0.96 | 7.03 | Village |  |
| parent | 161.62 | 45.12 | 3.85 | <LOD | <LOD | <LOD | Village |  |
| parent | <LOD | 32.28 | 18.43 | <LOD | <LOD | <LOD | Village |  |
| parent | 32.34 | 21.68 | 8.79 | <LOD | <LOD | <LOD | Village | **Hex** |
| parent | <LOD | <LOD | 4.03 | <LOD | <LOD | <LOD | Village | **River** |
| parent | 60.48 | <LOD | 2.04 | 3.01 | <LOD | 5.48 | Village | **Valley** |
| parent | 76.26 | <LOD | 4.93 | 1.20 | 8.31 | <LOD | Village |  |
| child | 151.02 | 37.92 | 2.84 | 9.59 | 1.92 | 5.28 | Farm |  |
| child | 66.38 | 11.62 | 2.45 | 1.34 | 2.53 | 5.74 | Farm |  |
| child | <LOD | <LOD | 3.93 | <LOD | <LOD | <LOD | Farm |  |
| child | 84.09 | 13.88 | 3.72 | <LOD | <LOD | <LOD | Farm |  |
| child | 95.42 | 11.98 | 1.32 | 2.71 | 1.32 | 15.00 | Farm |  |
| child | <LOD | 14.16 | <LOD | <LOD | <LOD | <LOD | Farm |  |
| child | 14.45 | <LOD | <LOD | 1.06 | <LOD | 15.25 | Farm |  |
| child | 34.75 | <LOD | 4.19 | <LOD | <LOD | 40.80 | Farm |  |
| child | 6.08 | <LOD | 5.46 | <LOD | <LOD | <LOD | Farm |  |
| child | <LOD | 13.00 | 4.19 | <LOD | <LOD | <LOD | Farm |  |
| child | <LOD | 8.72 | <LOD | <LOD | <LOD | <LOD | Village |  |
| child | 44.27 | <LOD | 2.16 | <LOD | 2.65 | 104.21 | Village |  |
| child | 99.87 | 7.78 | 3.68 | 98.39 | 1.50 | 8.76 | Village |  |
| child | 495.9 | 31.32 | 6.02 | 3.73 | <LOD | 128.19 | Village |  |
| child | <LOD | 41.38 | 3.97 | 1.80 | <LOD | 582.59 | Village |  |
| child | 8.85 | 48.43 | 5.90 | 1.92 | <LOD | <LOD | Village |  |
| child | <LOD | 28.13 | 40.42 | <LOD | <LOD | <LOD | Village |  |
| child | <LOD | <LOD | 4.10 | <LOD | <LOD | <LOD | Village |  |
| child | 83.73 | 31.20 | 4.21 | 3.19 | 2.46 | 13.85 | Village |  |

| **Participant** | **Chlorpyrifos** | **Cypermethrin** | **Deltamethrin** | **Diazinon** | **Malathion** | **Prothiofos** | **Location** | **Area** |
| --- | --- | --- | --- | --- | --- | --- | --- | --- |
| parent | 278.55 | <LOD | 2.53 | 1.92 | <LOD | 34.45 | Farm |  |
| parent | 6.86 | <LOD | 7.16 | <LOD | <LOD | 14.45 | Farm |  |
| parent | <LOD | <LOD | 2.34 | <LOD | <LOD | <LOD | Farm |  |
| parent | 157.4 | <LOD | 4.15 | 3.13 | <LOD | 21.62 | Farm |  |
| parent | 235.6 | <LOD | 352.28 | 2.16 | <LOD | 30.01 | Farm |  |
| parent | 26.74 | <LOD | 2.04 | 80.48 | <LOD | 48.61 | Farm |  |
| parent | 386.14 | <LOD | 3.73 | 12.46 | <LOD | 96.5 | Farm |  |
| parent | <LOD | <LOD | 1.44 | <LOD | <LOD | <LOD | Farm |  |
| parent | 121.92 | 15.9 | 2.28 | <LOD | <LOD | <LOD | Farm |  |
| parent | 8.01 | <LOD | <LOD | <LOD | <LOD | <LOD | Farm |  |
| parent | 11.44 | 1.08 | <LOD | <LOD | <LOD | <LOD | Village |  |
| parent | 61.08 | 3.67 | 2.77 | <LOD | <LOD | 53.25 | Village |  |
| parent | 25.90 | 20.90 | 1.26 | <LOD | <LOD | 14.21 | Village |  |
| parent | <LOD | 27.95 | 1.98 | <LOD | <LOD | <LOD | Village |  |
| parent | 60.84 | 45.60 | 2.16 | <LOD | <LOD | <LOD | Village |  |
| parent | 8.37 | <LOD | 2.89 | <LOD | <LOD | <LOD | Village |  |
| parent | 25.18 | <LOD | 3.79 | 11.08 | <LOD | <LOD | Village |  |
| parent | 26.14 | <LOD | 5.66 | <LOD | <LOD | <LOD | Village | **Grabouw** |
| parent | 16.44 | <LOD | 1.14 | <LOD | <LOD | <LOD | Village |  |
| child | 583.07 | <LOD | 3.25 | 2.59 | 1.44 | 72.89 | Farm |  |
| child | <LOD | 45.66 | 5.36 | <LOD | <LOD | <LOD | Farm |  |
| child | 658.49 | <LOD | 2.59 | 2.46 | <LOD | <LOD | Farm |  |
| child | 331.86 | <LOD | 7.53 | 6.56 | 3.19 | 45.9 | Farm |  |
| child | 409.63 | <LOD | 106.98 | 3.07 | <LOD | 56.68 | Farm |  |
| child | 36.62 | <LOD | 2.65 | 94.57 | <LOD | 69.45 | Farm |  |
| child | 584.57 | 18.07 | 6.26 | 9.57 | <LOD | 196.2 | Farm |  |
| child | 434.69 | 26.56 | 3.79 | 5.36 | <LOD | <LOD | Farm |  |
| child | <LOD | <LOD | 9.63 | <LOD | <LOD | <LOD | Farm |  |
| child | <LOD | 11.44 | 3.85 | <LOD | <LOD | <LOD | Farm |  |
| child | 6.44 | 38.91 | 3.73 | 1.86 | <LOD | <LOD | Village |  |
| child | 122.59 | <LOD | 3.19 | <LOD | <LOD | <LOD | Village |  |
| child | 74.15 | <LOD | 1.86 | <LOD | <LOD | <LOD | Village |  |
| child | 40.54 | 20.18 | 3.79 | 1.68 | 6.32 | 23.97 | Village |  |
| child | 136.2 | 85.18 | 5.9 | 3.79 | <LOD | 36.62 | Village |  |
| child | 44.03 | 18.19 | 13.91 | 1.08 | <LOD | 16.92 | Village |  |
| child | 64.33 | 8.01 | 10.04 | 19.03 | <LOD | 28.91 | Village |  |
| child | 254.21 | 88.61 | 43.91 | <LOD | <LOD | <LOD | Village |  |
| child | 211.26 | <LOD | 2.1 | 1.8 | <LOD | <LOD | Village |  |

# Table S3. OPs concentrations in household dust (ng/g).

| **Day** | **Area** | **Chlorpyrifos** | **Diazinon** | **Malathion** |
| --- | --- | --- | --- | --- |
| Day 1 | Hex River Valley | 3800.00 | 7.50 | <LOD* |
|  | Hex River Valley | 398.02 | 1396.04 | <LOD |
|  | Hex River Valley | 1235.29 | 6.27 | <LOD |
|  | Hex River Valley | 241.90 | 5.24 | <LOD |
|  | Hex River Valley | 492.40 | <iLOQ** | <LOD |
|  | Hex River Valley | 408.00 | 5.10 | <LOD |
|  | Grabouw | 990.29 | 10.87 | <LOD |
|  | Grabouw | 94.95 | 38.71 | <LOD |
|  | Grabouw | 221.21 | 14.95 | <LOD |
|  | Grabouw | 199.01 | 15.64 | <LOD |
|  | Grabouw | 364.08 | 4.37 | <LOD |
|  | Grabouw | 155.24 | 23.90 | <LOD |
| Day 7 | Hex River Valley | 425.49 | 12.84 | <LOD |
|  | Hex River Valley | 19528.30 | 15.47 | <LOD |
|  | Hex River Valley | 1904.76 | <iLOQ | <LOD |
|  | Hex River Valley | 189.32 | 6.41 | <LOD |
|  | Hex River Valley | 142.45 | 100.00 | <LOD |
|  | Hex River Valley | 86.99 | 36.12 | <LOD |
|  | Hex River Valley | 1847.62 | 7.71 | <LOD |
|  | Hex River Valley | 4900.00 | <iLOQ | <LOD |
|  | Hex River Valley | 3519.61 | <iLOQ | <LOD |
|  | Hex River Valley | 233.33 | <iLOQ | <LOD |
|  | Hex River Valley | 4601.94 | <iLOQ | <LOD |
|  | Hex River Valley | 255.34 | 4.47 | <LOD |
|  | Hex River Valley | 125.00 | <iLOQ | <LOD |
|  | Hex River Valley | 72.88 | 32.98 | <LOD |
|  | Hex River Valley | 410.89 | 14.75 | <LOD |
|  | Hex River Valley | 21.06 | <iLOQ | <LOD |
|  | Hex River Valley | 127.45 | 54.51 | <LOD |
|  | Hex River Valley | 370.00 | 15.30 | <LOD |
| Day 7 | Hex River Valley | <LOD | 1682.69 | <LOD |
|  | Grabouw | 975.00 | 4.80 | <LOD |
|  | Grabouw | 180.95 | 4.95 | <LOD |
|  | Grabouw | 365.69 | <iLOQ | <LOD |
|  | Grabouw | 1098.04 | 10.88 | <LOD |
|  | Grabouw | 203.96 | 72.57 | <LOD |
|  | Grabouw | 406.80 | 6.02 | <LOD |
|  | Grabouw | 3567.31 | 17.79 | <LOD |
|  | Grabouw | 268.18 | 37.42 | 149.55 |
|  | Grabouw | <LOD | 11.19 | 43.86 |
|  | Grabouw | 86.08 | 73.73 | <LOD |
|  | Grabouw | 95.49 | 40.88 | <LOD |
|  | Grabouw | 113.33 | 2209.52 | <LOD |
|  | Grabouw | 133.01 | 86.89 | <LOD |
|  | Grabouw | 60.70 | 25.70 | <LOD |
|  | Grabouw | 480.00 | <iLOQ | <LOD |
|  | Grabouw | 4701.92 | <iLOQ | <LOD |
|  | Grabouw | 465.71 | <iLOQ | <LOD |
|  | Grabouw | 1067.31 | <iLOQ | <LOD |
|  | Grabouw | 947.62 | <iLOQ | <LOD |

* LOD – Limit of detection

** iLOQ – Instrumental limit of quantification

# Table S4. Correlation matrix showing the Spearman rank correlations (R*s* upper diagonal, non-italic) and respective *p*-values (lower diagonal, italic) between the levels of organophosphates (OPs) and pyrethroids (PYRs) measured in urine, wristbands and household dust measured at day 1 and day 7.

|  | TCPy day 1 (OP) | TCPy day 7 (OP) | Chlorpyrifos dust day 1 (OP) | Chlorpyrifos dust day 7 (OP) | Chlorpyrifos wristband child (OP) | Chlorpyrifos wristband guardian (OP) | IMPy day 1 (OP) | IMPy day 7 (OP) | Diazinon dust day 1 (OP) | Diazinon dust day 7 (OP) |
| --- | --- | --- | --- | --- | --- | --- | --- | --- | --- | --- |
| TCPy day 1 (OP) | *** | 0.41 | 0.31 | 0.24 | 0.27 | 0.20 | 0.07 | -0.32 | 0.15 | -0.04 |
| TCPy day 7 (OP) | *0.01* | *** | 0.07 | 0.44 | -0.03 | 0.08 | -0.15 | -0.26 | -0.29 | -0.35 |
| Chlorpyrifos dust day 1 (OP) | *0.33* | *0.83* | *** | 0.69 | 0.07 | 0.17 | 0.38 | -0.28 | -0.50 | -0.43 |
| Chlorpyrifos dust day 7 (OP) | *0.14* | *0.01* | *0.02* | *** | 0.17 | 0.16 | 0.07 | 0.11 | -0.46 | -0.52 |
| Chlorpyrifos wristband child (OP) | *0.10* | *0.88* | *0.84* | *0.30* | *** | 0.40 | -0.25 | 0.27 | 0.50 | -0.29 |
| Chlorpyrifos wristband guardian (OP) | *0.23* | *0.65* | *0.60* | *0.32* | *0.01* | *** | -0.05 | 0.11 | -0.20 | -0.29 |
| IMPy day 1 (OP) | *0.66* | *0.37* | *0.23* | *0.69* | *0.13* | *0.77* | *** | 0.01 | -0.10 | 0.11 |
| IMPy day 7 (OP) | *0.05* | *0.11* | *0.38* | *0.50* | *0.10* | *0.51* | *0.96* | *** | -0.01 | -0.02 |
| Diazinon dust day 1 (OP) | *0.64* | *0.35* | *0.10* | *0.13* | *0.10* | *0.54* | *0.75* | *0.97* | *** | 0.32 |
| Diazinon dust day 7 (OP) | *0.82* | *0.03* | *0.17* | *0.00* | *0.08* | *0.08* | *0.51* | *0.89* | *0.31* | *** |
| Diazinon wristband child (OP) | *0.01* | *0.47* | *0.72* | *0.36* | *0.00* | *0.03* | *0.81* | *0.12* | *0.01* | *0.52* |
| Diazinon wristband guardian (OP) | *0.09* | *0.63* | *0.65* | *0.45* | *0.09* | *0.00* | *0.44* | *0.16* | *0.22* | *0.29* |
| Malathion dust day 7 (OP) | *0.62* | *0.87* | *0.50* | *0.24* | *0.79* | *0.36* | *0.24* | *0.37* | *0.68* | *0.42* |
| Malathion wristband child (OP) | *0.99* | *0.90* | *0.27* | *0.91* | *0.09* | *0.21* | *0.71* | *0.27* | *0.28* | *0.88* |
| Malathion wristband guardian (OP) | *0.87* | *0.66* | *0.89* | *0.56* | *0.70* | *0.15* | *0.09* | *0.60* | *0.11* | *0.94* |
| DEP day 1 (OP) | *0.00* | *0.07* | *0.42* | *0.31* | *0.81* | *0.56* | *0.31* | *0.40* | *0.51* | *0.78* |
| DEP day 7 (OP) | *0.02* | *0.00* | *0.60* | *0.53* | *0.49* | *0.92* | *0.55* | *0.08* | *0.60* | *0.35* |
| DETP day 1 (OP) | *0.01* | *0.58* | *0.03* | *0.19* | *0.14* | *0.87* | *0.10* | *0.48* | *0.42* | *0.93* |
| DETP day 7 (OP) | *0.03* | *0.02* | *0.64* | *0.01* | *0.23* | *0.39* | *0.57* | *0.96* | *0.24* | *0.46* |
| DMP day 1 (OP) | *0.59* | *0.76* | *0.09* | *0.38* | *0.46* | *0.74* | *0.67* | *0.61* | *0.06* | *0.68* |
| DMP day 7 (OP) | *0.06* | *0.74* | *0.68* | *0.40* | *0.03* | *0.39* | *0.55* | *0.62* | *0.07* | *0.07* |
| DMTP day 1 (OP) | *0.50* | *0.14* | *0.57* | *0.87* | *0.27* | *0.59* | *0.51* | *0.67* | *0.20* | *0.43* |
| DMTP day 7 (OP) | *0.88* | *0.76* | *0.78* | *0.55* | *0.37* | *0.98* | *0.40* | *0.99* | *0.50* | *0.34* |
| Prothiofos wristband child (OP) | *0.01* | *0.18* | *0.77* | *0.21* | *0.02* | *0.02* | *0.43* | *0.24* | *0.18* | *0.64* |
| Prothiofos wristband guardian (OP) | *0.14* | *0.45* | *0.55* | *0.10* | *0.08* | *0.00* | *0.97* | *0.42* | *0.24* | *0.06* |
| 3-PBA day 1 (PYR) | *0.78* | *0.39* | *0.92* | *0.35* | *0.16* | *0.42* | *0.45* | *0.13* | *0.59* | *0.32* |
| 3-PBA day 7 (PYR) | *0.80* | *0.84* | *0.75* | *0.09* | *0.51* | *0.39* | *0.09* | *0.27* | *0.65* | *0.29* |
| DCCA day 1 (PYR) | *0.53* | *0.80* | *0.47* | *0.85* | *0.14* | *0.19* | *0.47* | *0.55* | *0.08* | *0.07* |
| DCCA day 7 (PYR) | *0.75* | *0.98* | *0.28* | *0.75* | *0.63* | *0.72* | *0.88* | *0.76* | *0.21* | *0.77* |
| Cypermethrin wristband child (PYR) | *0.96* | *0.49* | *0.48* | *0.38* | *0.85* | *0.28* | *0.78* | *0.21* | *0.48* | *0.05* |
| Cypermethrin wristband guardian (PYR) | *0.05* | *0.51* | *0.40* | *0.43* | *0.60* | *0.36* | *0.58* | *0.68* | *0.36* | *0.46* |
| Deltamethrin wristband child (PYR) | *0.02* | *0.83* | *0.78* | *0.24* | *0.85* | *0.26* | *0.46* | *0.38* | *0.57* | *0.60* |
| Deltamethrin wristband guardian (PYR) | *0.00* | *0.33* | *0.62* | *0.61* | *0.33* | *0.03* | *1.00* | *0.03* | *0.45* | *0.76* |

Table S4. (cont.)

|  | Diazinon wristband child (OP) | Diazinon wristband guardian (OP) | Malathion dust day 7 (OP) | Malathion wristband child (OP) | Malathion wristband guardian (OP) | DEP day 1 (OP) | DEP day 7 (OP) | DETP day 1 (OP) | DETP day 7 (OP) | DMP day 1 (OP) | DMP day 7 (OP) | DMTP day 1 (OP) | DMTP day 7 (OP) |
| --- | --- | --- | --- | --- | --- | --- | --- | --- | --- | --- | --- | --- | --- |
| TCPy day 1 (OP) | 0.44 | 0.28 | 0.08 | 0.00 | -0.03 | 0.53 | 0.38 | 0.43 | 0.35 | -0.09 | 0.31 | -0.11 | -0.03 |
| TCPy day 7 (OP) | 0.12 | 0.08 | -0.03 | 0.02 | -0.07 | 0.30 | 0.60 | 0.09 | 0.38 | -0.05 | -0.06 | -0.24 | -0.05 |
| Chlorpyrifos dust day 1 (OP) | -0.12 | -0.15 | -0.22 | -0.35 | 0.04 | 0.26 | 0.17 | 0.64 | -0.15 | 0.51 | -0.13 | 0.18 | -0.09 |
| Chlorpyrifos dust day 7 (OP) | 0.15 | 0.13 | -0.19 | 0.02 | -0.10 | 0.17 | 0.11 | 0.22 | 0.42 | -0.15 | -0.14 | 0.03 | -0.10 |
| Chlorpyrifos wristband child (OP) | 0.61 | 0.28 | -0.04 | 0.28 | 0.06 | 0.04 | 0.11 | 0.25 | 0.20 | -0.12 | 0.35 | -0.18 | 0.15 |
| Chlorpyrifos wristband guardian (OP) | 0.35 | 0.57 | -0.15 | 0.21 | 0.24 | 0.10 | 0.02 | 0.03 | 0.14 | -0.05 | 0.14 | -0.09 | -0.01 |
| IMPy day 1 (OP) | 0.04 | 0.13 | -0.19 | 0.06 | 0.28 | 0.17 | -0.10 | 0.27 | -0.10 | 0.07 | -0.10 | 0.11 | -0.14 |
| IMPy day 7 (OP) | 0.26 | 0.23 | -0.15 | 0.18 | -0.09 | -0.14 | -0.29 | -0.12 | 0.01 | 0.09 | 0.08 | 0.07 | 0.00 |
| Diazinon dust day 1 (OP) | 0.70 | 0.39 | 0.13 | 0.34 | 0.48 | -0.21 | 0.17 | -0.26 | 0.37 | -0.56 | 0.54 | -0.40 | 0.22 |
| Diazinon dust day 7 (OP) | -0.11 | -0.18 | 0.13 | -0.03 | 0.01 | 0.05 | -0.16 | 0.01 | -0.12 | 0.07 | 0.30 | -0.13 | 0.16 |
| Diazinon wristband child (OP) | *** | 0.58 | -0.10 | 0.32 | 0.10 | 0.29 | 0.21 | 0.26 | 0.25 | 0.14 | 0.28 | -0.16 | -0.18 |
| Diazinon wristband guardian (OP) | *0.00* | *** | -0.17 | 0.27 | 0.31 | 0.15 | -0.13 | 0.06 | 0.06 | -0.09 | 0.08 | 0.11 | -0.13 |
| Malathion dust day 7 (OP) | *0.53* | *0.32* | *** | -0.13 | -0.11 | -0.14 | 0.18 | -0.07 | -0.09 | -0.05 | -0.13 | 0.12 | -0.01 |
| Malathion wristband child (OP) | *0.05* | *0.10* | *0.44* | *** | 0.47 | -0.18 | -0.10 | -0.06 | 0.06 | -0.09 | 0.05 | -0.26 | 0.09 |
| Malathion wristband guardian (OP) | *0.55* | *0.06* | *0.51* | *0.00* | *** | 0.00 | 0.07 | 0.03 | -0.03 | -0.05 | 0.14 | -0.10 | 0.20 |
| DEP day 1 (OP) | *0.08* | *0.37* | *0.39* | *0.29* | *0.99* | *** | 0.23 | 0.54 | 0.35 | -0.01 | 0.30 | 0.11 | 0.11 |
| DEP day 7 (OP) | *0.21* | *0.43* | *0.28* | *0.55* | *0.66* | *0.17* | *** | 0.21 | 0.44 | 0.09 | 0.24 | -0.24 | -0.03 |
| DETP day 1 (OP) | *0.12* | *0.72* | *0.69* | *0.74* | *0.85* | *0.00* | *0.21* | *** | 0.42 | -0.09 | 0.23 | 0.20 | 0.19 |
| DETP day 7 (OP) | *0.13* | *0.72* | *0.59* | *0.73* | *0.88* | *0.03* | *0.01* | *0.01* | *** | -0.17 | 0.44 | -0.10 | 0.22 |
| DMP day 1 (OP) | *0.40* | *0.57* | *0.78* | *0.58* | *0.74* | *0.93* | *0.61* | *0.60* | *0.31* | *** | -0.12 | -0.07 | -0.39 |
| DMP day 7 (OP) | *0.09* | *0.64* | *0.44* | *0.75* | *0.41* | *0.07* | *0.15* | *0.17* | *0.01* | *0.48* | *** | -0.36 | 0.36 |
| DMTP day 1 (OP) | *0.35* | *0.52* | *0.49* | *0.12* | *0.54* | *0.50* | *0.15* | *0.24* | *0.53* | *0.69* | *0.03* | *** | 0.08 |
| DMTP day 7 (OP) | *0.29* | *0.45* | *0.94* | *0.59* | *0.23* | *0.50* | *0.86* | *0.24* | *0.19* | *0.02* | *0.03* | *0.64* | *** |
| Prothiofos wristband child (OP) | *0.00* | *0.00* | *0.19* | *0.02* | *0.29* | *0.05* | *0.62* | *0.48* | *0.28* | *0.38* | *0.53* | *0.70* | *0.25* |
| Prothiofos wristband guardian (OP) | *0.36* | *0.00* | *0.27* | *0.11* | *0.08* | *0.46* | *0.67* | *0.51* | *0.13* | *0.06* | *0.15* | *0.93* | *0.74* |
| 3-PBA day 1 (PYR) | *0.69* | *0.90* | *0.90* | *0.92* | *0.74* | *0.25* | *0.57* | *0.97* | *0.75* | *0.58* | *0.03* | *0.00* | *0.50* |
| 3-PBA day 7 (PYR) | *0.12* | *0.12* | *0.38* | *0.74* | *0.48* | *0.34* | *0.72* | *0.36* | *0.14* | *0.69* | *0.22* | *0.07* | *0.06* |
| DCCA day 1 (PYR) | *0.07* | *0.49* | *0.48* | *0.48* | *0.66* | *0.94* | *0.82* | *0.92* | *0.80* | *0.89* | *0.00* | *0.00* | *0.60* |
| DCCA day 7 (PYR) | *0.15* | *0.05* | *0.93* | *0.53* | *0.31* | *0.66* | *0.60* | *0.23* | *0.37* | *0.53* | *0.85* | *0.30* | *0.04* |
| Cypermethrin wristband child (PYR) | *0.32* | *0.06* | *0.44* | *0.84* | *0.62* | *0.44* | *0.87* | *0.93* | *0.98* | *0.56* | *0.95* | *0.97* | *0.94* |
| Cypermethrin wristband guardian (PYR) | *0.81* | *0.22* | *0.19* | *0.33* | *0.72* | *0.56* | *0.15* | *0.63* | *0.23* | *0.00* | *0.19* | *0.35* | *0.25* |
| Deltamethrin wristband child (PYR) | *0.58* | *0.81* | *0.38* | *0.19* | *0.38* | *0.11* | *0.97* | *0.94* | *0.77* | *0.59* | *0.20* | *0.92* | *0.47* |
| Deltamethrin wristband guardian (PYR) | *0.16* | *0.05* | *0.06* | *0.49* | *0.22* | *0.23* | *0.39* | *0.67* | *0.84* | *0.84* | *0.26* | *0.60* | *0.33* |

Table S4. (cont.)

|  | Prothiofos wristband child (OP) | Prothiofos wristband guardian (OP) | 3-PBA day 1 (PYR) | 3-PBA day 7 (PYR) | DCCA day 1 (PYR) | DCCA day 7 (PYR) | Cypermethrin wristband child (PYR) | Cypermethrin wristband guardian (PYR) | Deltamethrin wristband child (PYR) | Deltamethrin wristband guardian (PYR) |
| --- | --- | --- | --- | --- | --- | --- | --- | --- | --- | --- |
| TCPy day 1 (OP) | 0.40 | 0.24 | 0.05 | -0.04 | -0.10 | -0.05 | -0.01 | -0.32 | 0.38 | 0.46 |
| TCPy day 7 (OP) | 0.22 | 0.13 | 0.14 | -0.03 | 0.04 | 0.00 | -0.12 | 0.11 | 0.04 | 0.16 |
| Chlorpyrifos dust day 1 (OP) | -0.09 | -0.19 | 0.03 | -0.10 | 0.23 | 0.34 | -0.22 | 0.27 | -0.09 | 0.16 |
| Chlorpyrifos dust day 7 (OP) | 0.21 | 0.27 | -0.15 | -0.28 | 0.03 | -0.05 | -0.15 | 0.13 | -0.19 | -0.08 |
| Chlorpyrifos wristband child (OP) | 0.38 | 0.29 | -0.24 | -0.11 | -0.24 | 0.08 | -0.03 | -0.09 | 0.03 | 0.16 |
| Chlorpyrifos wristband guardian (OP) | 0.39 | 0.50 | -0.13 | -0.14 | -0.22 | -0.06 | -0.18 | 0.15 | 0.19 | 0.36 |
| IMPy day 1 (OP) | 0.13 | 0.01 | 0.13 | 0.28 | 0.12 | 0.03 | -0.05 | 0.09 | -0.12 | 0.00 |
| IMPy day 7 (OP) | 0.19 | 0.13 | -0.25 | -0.18 | -0.10 | 0.05 | -0.21 | -0.07 | -0.15 | -0.35 |
| Diazinon dust day 1 (OP) | 0.41 | 0.37 | -0.17 | -0.15 | -0.53 | -0.39 | 0.22 | -0.29 | -0.18 | 0.24 |
| Diazinon dust day 7 (OP) | -0.08 | -0.30 | -0.17 | 0.18 | -0.30 | 0.05 | 0.32 | -0.12 | 0.09 | -0.05 |
| Diazinon wristband child (OP) | 0.56 | 0.15 | -0.07 | -0.26 | -0.30 | -0.24 | 0.17 | 0.04 | 0.09 | 0.23 |
| Diazinon wristband guardian (OP) | 0.52 | 0.54 | 0.02 | -0.26 | -0.11 | -0.32 | -0.31 | -0.20 | 0.04 | 0.32 |
| Malathion dust day 7 (OP) | -0.22 | -0.18 | 0.02 | 0.15 | 0.12 | 0.02 | -0.13 | -0.22 | -0.15 | -0.31 |
| Malathion wristband child (OP) | 0.38 | 0.26 | -0.02 | 0.06 | -0.12 | -0.10 | -0.03 | 0.16 | -0.22 | 0.12 |
| Malathion wristband guardian (OP) | 0.18 | 0.29 | 0.06 | 0.12 | 0.07 | 0.17 | -0.08 | 0.06 | -0.15 | 0.21 |
| DEP day 1 (OP) | 0.32 | -0.12 | 0.19 | 0.16 | -0.01 | 0.07 | 0.13 | -0.10 | 0.27 | 0.20 |
| DEP day 7 (OP) | 0.08 | 0.07 | 0.10 | -0.06 | 0.04 | 0.09 | 0.03 | 0.24 | 0.01 | 0.14 |
| DETP day 1 (OP) | 0.12 | -0.11 | -0.01 | 0.15 | 0.02 | 0.20 | -0.02 | -0.08 | 0.01 | 0.07 |
| DETP day 7 (OP) | 0.18 | 0.25 | -0.05 | -0.25 | 0.04 | -0.15 | 0.00 | 0.20 | 0.05 | 0.03 |
| DMP day 1 (OP) | 0.15 | -0.31 | 0.09 | -0.07 | 0.02 | -0.10 | 0.10 | 0.49 | -0.09 | 0.03 |
| DMP day 7 (OP) | 0.10 | 0.24 | -0.36 | -0.20 | -0.46 | -0.03 | -0.01 | -0.22 | 0.21 | 0.19 |
| DMTP day 1 (OP) | -0.06 | -0.01 | 0.50 | 0.30 | 0.62 | 0.17 | -0.01 | -0.15 | -0.02 | -0.09 |
| DMTP day 7 (OP) | -0.19 | 0.06 | 0.11 | 0.31 | 0.09 | 0.33 | 0.01 | -0.19 | -0.12 | -0.16 |
| Prothiofos wristband child (OP) | *** | 0.37 | 0.05 | 0.11 | -0.08 | -0.02 | -0.03 | 0.14 | 0.15 | 0.46 |
| Prothiofos wristband guardian (OP) | *0.02* | *** | -0.13 | -0.23 | -0.01 | -0.06 | -0.43 | -0.19 | -0.08 | 0.29 |
| 3-PBA day 1 (PYR) | *0.76* | *0.43* | *** | 0.45 | 0.72 | 0.15 | 0.16 | 0.08 | 0.10 | 0.03 |
| 3-PBA day 7 (PYR) | *0.52* | *0.16* | *0.01* | *** | 0.34 | 0.56 | 0.05 | 0.03 | -0.05 | 0.08 |
| DCCA day 1 (PYR) | *0.64* | *0.96* | *0.00* | *0.04* | *** | 0.16 | 0.12 | 0.06 | -0.04 | -0.07 |
| DCCA day 7 (PYR) | *0.89* | *0.73* | *0.36* | *0.00* | *0.35* | *** | -0.12 | -0.09 | 0.02 | -0.03 |
| Cypermethrin wristband child (PYR) | *0.88* | *0.01* | *0.32* | *0.77* | *0.47* | *0.49* | *** | 0.28 | 0.30 | 0.18 |
| Cypermethrin wristband guardian (PYR) | *0.40* | *0.25* | *0.62* | *0.85* | *0.74* | *0.61* | *0.09* | *** | -0.32 | 0.02 |
| Deltamethrin wristband child (PYR) | *0.36* | *0.65* | *0.57* | *0.76* | *0.83* | *0.91* | *0.06* | *0.05* | *** | 0.49 |
| Deltamethrin wristband guardian (PYR) | *0.00* | *0.07* | *0.88* | *0.63* | *0.66* | *0.84* | *0.27* | *0.92* | *0.00* | *** |

# Table S5. Parameters used to calculate the estimated daily intake (EDI) using the urine biomonitoring levels.

| **Pesticide** | **Acceptable daily intake (µg/kg/day)*** | **Biomarker** | **Child daily urinary excretion volume (L/day)**** | **Pesticide molecular weight (g/mol)** | **Metabolite molecular weight (g/mol)** | **Urinary excretion factor** |
| --- | --- | --- | --- | --- | --- | --- |
| Chlorpyrifos | 1 | TCPy | Excretion volume | 350.6 | 198.4 | 0.7  (Nolan et al., 1984) |
| Chlorpyrifos | 1 | ∑DEPs | depending on the age: | 350.6 | 222.2 | 0.7  (Nolan et al., 1984) |
| Diazinon | 0.2 | IMPy | 9-12 y: 0.77 L/day | 304.4 | 152.2 | 0.6  (Garfitt et al., 2002) |
| Parathion | 0.6 | PNP | 13-15 y: 1.07 L/day | 291.3 | 139.11 | 0.36 (Morgan et al., 1977) |
| Deltamethrin | 10 | 3-PBA |  | 505.2 | 214.22 | 0.24  (Sams & Jones, 2012) |
| Cypermethrin | 5 | DCCA |  | 416.3 | 209.1 | 0.36  (Ratelle et al., 2015; Woollen et al., 1992) |

*Acceptable daily intake established by EFSA and European Commission (EFSA, 2014).

** The mean values of child urinary excreted volume for each age group were taken from the systematic literature review by Beckford et al. (2020).

| **Estimated Daily Intake** | **µg/kg/day** | **Day 1** | | | | | **Day 7** | | | | |
| --- | --- | --- | --- | --- | --- | --- | --- | --- | --- | --- | --- |
| Pesticide | Biomarker | EDI_25_ | EDI_50_ | EDI_75_ | EDI_95_ | EDI_MAX_ | EDI_25_ | EDI_50_ | EDI_75_ | EDI_95_ | EDI_MAX_ |
| Chlorpyrifos | TCPy | 0.03 | 0.08 | 0.15 | 0.47 | 4.63 | 0.005 | 0.02 | 0.04 | 0.12 | 1.01 |
| Chlorpyrifos | ∑DEPs | 0.09 | 0.13 | 0.22 | 0.50 | 1.39 | 0.09 | 0.14 | 0.23 | 0.78 | 4.99 |
| Diazinon | IMPy | 0.01 | 0.03 | 0.13 | 0.63 | 2.23 | 0.003 | 0.01 | 0.02 | 0.17 | 0.68 |
| Parathion | PNP | 0.01 | 0.02 | 0.04 | 0.09 | 0.855 | 0.001 | 0.04 | 0.13 | 0.30 | 0.35 |
| Deltamethrin | 3-PBA | 0.11 | 0.17 | 0.27 | 0.53 | 1.79 | 0.08 | 0.26 | 0.39 | 1.07 | 1.79 |
| Cypermethrin | DCCA | 0.05 | 0.09 | 0.13 | 0.24 | 0.728 | 0.04 | 0.12 | 0.18 | 0.29 | 0.36 |
| **Hazard Quotient** |  |  |  |  |  |  |  |  |  |  |  |
| Pesticide | Biomarker | HQ_25_ | HQ_50_ | HQ_75_ | HQ_95_ | HQ_MAX_ | HQ_25_ | HQ_50_ | HQ_75_ | HQ_95_ | HQ_MAX_ |
| Chlorpyrifos | TCPy | 0.03 | 0.08 | 0.15 | 0.47 | 4.63 | 0.005 | 0.02 | 0.04 | 0.12 | 1.01 |
| Chlorpyrifos | ∑DEPs | 0.09 | 0.13 | 0.22 | 0.50 | 1.39 | 0.09 | 0.14 | 0.23 | 0.78 | 4.99 |
| Diazinon | IMPy | 0.04 | 0.16 | 0.65 | 3.16 | 11.14 | 0.02 | 0.05 | 0.11 | 0.86 | 3.39 |
| Parathion | PNP | 0.01 | 0.03 | 0.06 | 0.15 | 1.43 | 0.001 | 0.06 | 0.22 | 0.50 | 0.58 |
| Deltamethrin | 3-PBA | 0.01 | 0.02 | 0.03 | 0.05 | 0.12 | 0.01 | 0.03 | 0.04 | 0.11 | 0.18 |
| Cypermethrin | DCCA | 0.01 | 0.02 | 0.03 | 0.05 | 0.14 | 0.01 | 0.02 | 0.04 | 0.06 | 0.07 |
| **Hazard Index** |  |  |  |  |  |  |  |  |  |  |  |
| Pesticide group | MoA* | HI_25_ | HI_50_ | HI_75_ | HI_95_ | HI_MAX_ | HI_25_ | HI_50_ | HI_75_ | HI_95_ | HI_MAX_ |
| Organophosphates | AChE inhibitors | 0.19 | 0.37 | 0.87 | 5.29 | 11.61 | 0.08 | 0.19 | 0.36 | 1.62 | 3.41 |
| Pyrethroids | Sodium channel modulators | 0.02 | 0.03 | 0.05 | 0.11 | 0.27 | 0.02 | 0.05 | 0.07 | 0.15 | 0.22 |

# Table S6. Descriptive statistics of EDI (µg/kg/day), HQ and HI (unitless) calculated for each pesticide and pesticide group at Day 1 and Day 7.

*MoA – Mode of Action; AChE - Acetylcholinesterase

# Table S7. Linear mixed effect models to study the effect of area and location on the overall log standardized concentrations and models stratified per matrix. The ID was taken as a random effect

| **Overall model (log standardized values – unitless)** |  |  |  |
| --- | --- | --- | --- |
| Predictor | β-estimate | 95% CI | *p*-value |
| Location ^a^: Village | -0.08 | -0.29 – 0.14 | 0.49 |
| Area ^b^: Hex River Valley | 0.09 | -0.13 – 0.31 | 0.40 |
| Sex ^c^: Male | -0.03 | -0.25 – 0.19 | 0.78 |
|  |  |  |  |
| **Urine biomarkers (log µg/g creatinine)** |  |  |  |
| Predictor | β-estimate | 95% CI | *p*-value |
| Location: Village | -0.17 | -0.46 – 0.11 | 0.23 |
| Area: Hex River Valley | 0.36 | 0.08 – 0.65 | 0.02 |
| Sex: Male | 0.03 | -0.26 – 0.33 | 0.81 |
|  |  |  |  |
| **Wristbands (log ng/g wristband)** |  |  |  |
| Predictor | β-estimate | 95% CI | *p*-value |
| Location: Village | 0.38 | -0.64 – 1.408 | 0.45 |
| Area: Hex River Valley | -0.86 | -1.88 – 0.17 | 0.09 |
| Sex: Male | 0.07 | -0.97 – 1.11 | 0.88 |
|  |  |  |  |
| **Household dust (log ng/g dust)** |  |  |  |
| Predictor | β-estimate | 95% CI | *p*-value |
| Location: Village | 0.3 | -0.71 – 1.32 | 0.55 |
| Area: Hex River Valley | 0.01 | -1.01 – 1.02 | 0.99 |

^a^ Farm was taken as the reference value; ^b^ Grabouw was taken as the reference value; ^c^ Female was taken as the reference value.

Unadjusted and adjusted intraclass correlation coefficient (ICC) from LME models.

|  | Intraclass correlation coefficient (ICC) | |
| --- | --- | --- |
|  | Unadjusted | Adjusted for predictors |
| Overall | 0.07 | 0.07 |
| Urine | 0.00 | 0.00 |
| Wristband | 0.16 | 0.16 |
| Dust* |  | |

* For dust samples the variance of random effect was 0 and we could not calculate the ICC.

# Figure S1. Correlation matrix showing the Spearman rank correlations (R*s*) between the levels of organophosphates (OPs) and pyrethroids (PYRs) in urine, wristbands and dust measured at day 1 and day 7. For *p*-values consult Table S6.


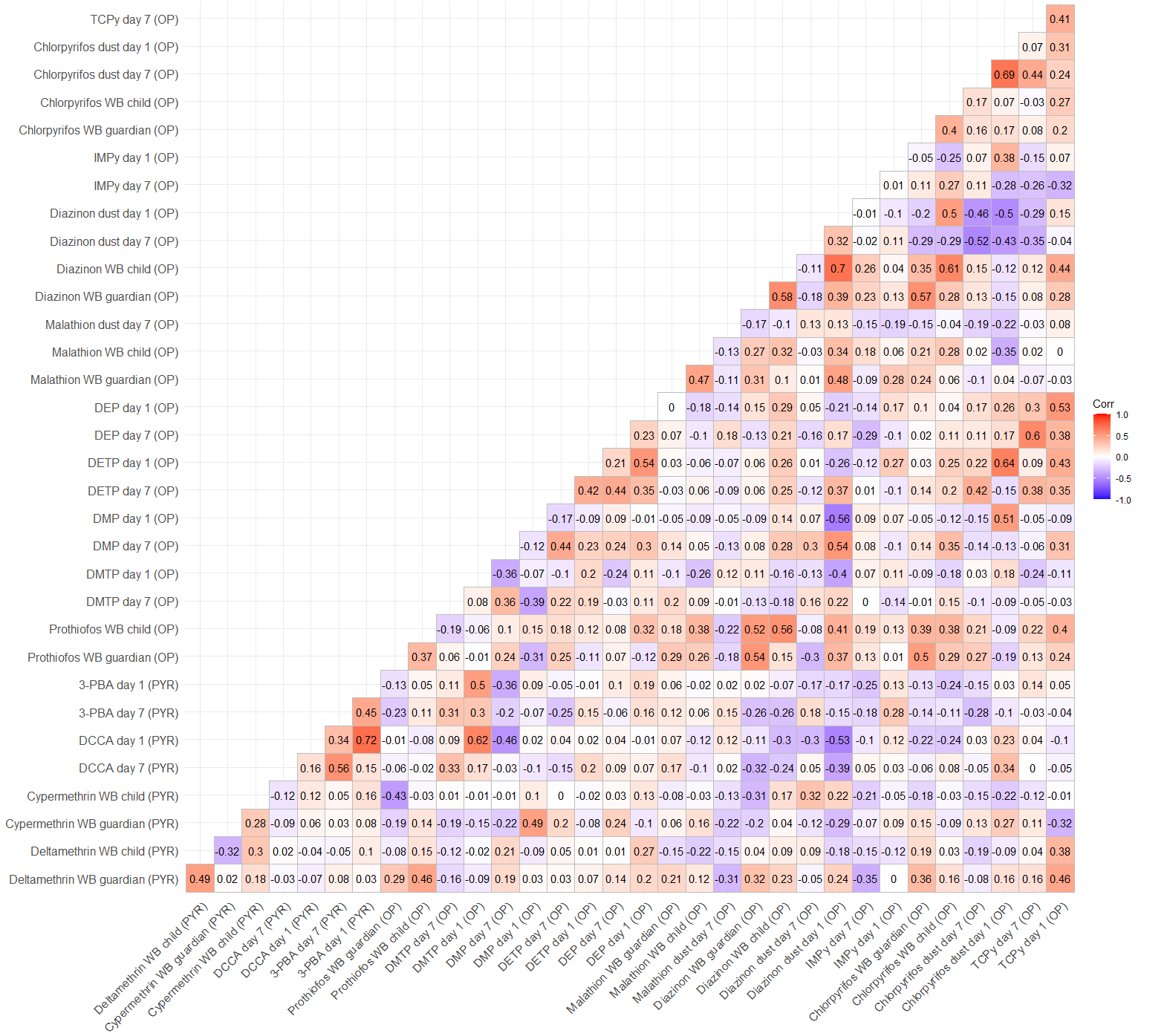


#
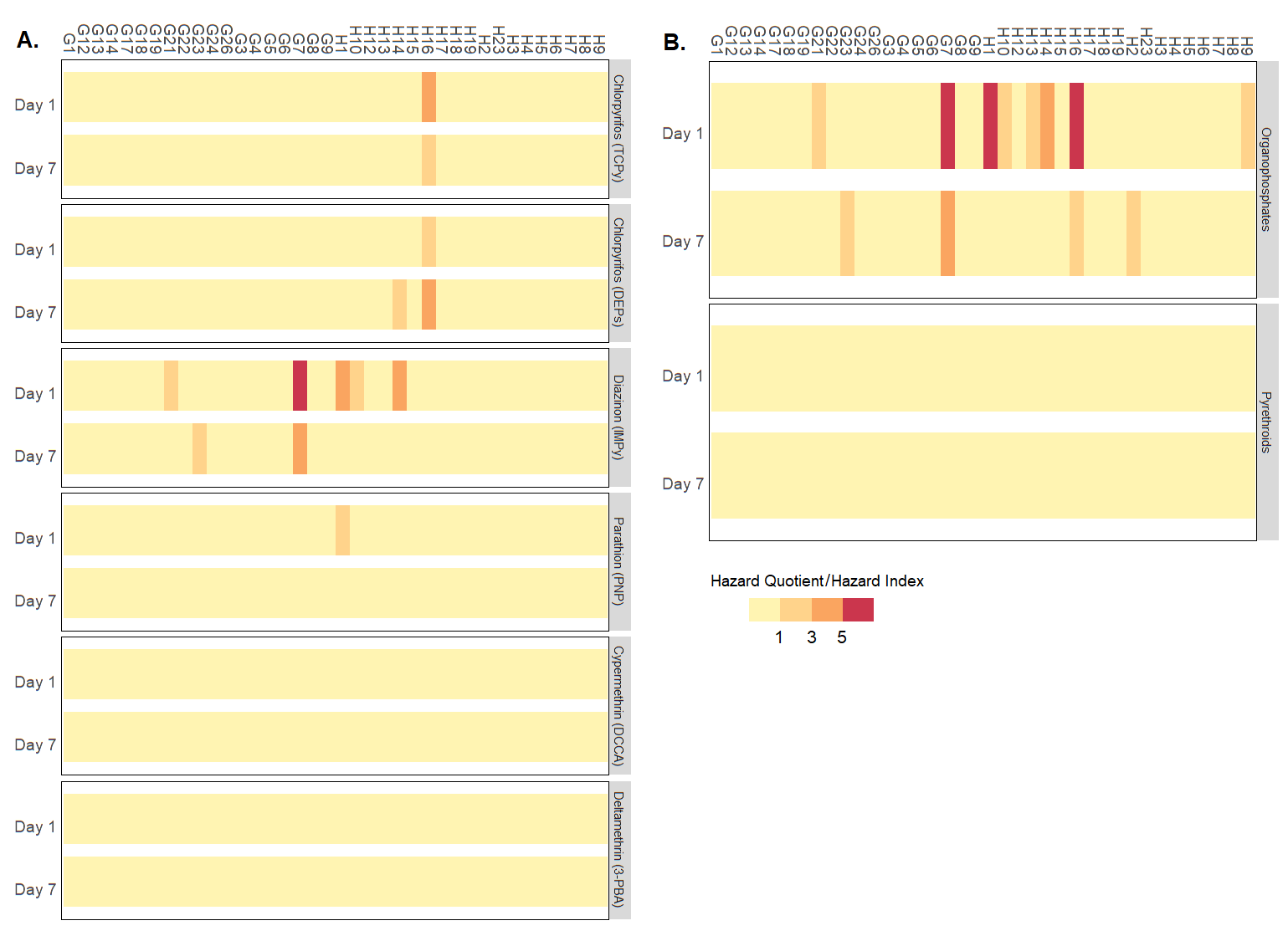
Figure S2. Estimated Hazard Quotient (A) and Hazard Index (B) for each pesticide and pesticide group, respectively, stratified per day. A value above one (range from light orange to red, i.e., from lower to higher values) indicates a possible risk of health effects due to exposure to a specific pesticide (A) or pesticide group (B).

# **References**

Beckford, K., Grimes, C. A., Margerison, C., Riddell, L. J., Skeaff, S. A., West, M. L., & Nowson, C. A. (2020). A systematic review and meta-analysis of 24-h urinary output of children and adolescents: impact on the assessment of iodine status using urinary biomarkers. *European Journal of Nutrition*, *59*(7), 3113–3131. https://doi.org/10.1007/s00394-019-02151-w

EFSA. *EU Pesticides Database*. Retrieved July 28, 2022, from https://food.ec.europa.eu/plants/pesticides/eu-pesticides-database_en

EFSA. (2014). Conclusion on the peer review of the pesticide human health risk assessment of the active substance chlorpyrifos. In *EFSA Journal* (Vol. 12, Issue 4). https://doi.org/10.2903/j.efsa.2014.3640

Garfitt, S. J., Jones, K., Mason, H. J., & Cocker, J. (2002). Exposure to the organophosphate diazinon: Data from a human volunteer study with oral and dermal doses. *Toxicology Letters*, *134*(1–3), 105–113. https://doi.org/10.1016/S0378-4274(02)00178-9

Morgan, D. P., Hetzler, H. L., Slach, E. F., & Lin, L. I. (1977). Urinary excretion of paranitrophenol and alkyl phosphates following ingestion of methyl or ethyl parathion by human subjects. *Archives of Environmental Contamination and Toxicology*, *6*(1), 159–173. https://doi.org/10.1007/BF02097758

Nolan, R. J., Rick, D. L., Freshour, N. L., & Saunders, J. H. (1984). Chlorpyrifos: Pharmacokinetics in human volunteers. *Toxicology and Applied Pharmacology*, *73*(1), 8–15. https://doi.org/10.1016/0041-008X(84)90046-2

Ratelle, M., Coté, J., & Bouchard, M. (2015). Time profiles and toxicokinetic parameters of key biomarkers of exposure to cypermethrin in orally exposed volunteers compared with previously available kinetic data following permethrin exposure. *Journal of Applied Toxicology*, *35*, 1586–1593. https://doi.org/10.1002/jat.3124

Sams, C., & Jones, K. (2012). Biological monitoring for exposure to deltamethrin: A human oral dosing study and background levels in the UK general population. *Toxicology Letters*, *213*(1), 35–38. https://doi.org/10.1016/j.toxlet.2011.04.014

Woollen, B. H., Marsh, J. R., Laird, W. J. D., & Lesser, J. E. (1992). The metabolism of cypermethrin in man: Differences in urinary metabolite profiles following oral and dermal administration. *Xenobiotica*, *22*(8), 983–991. https://doi.org/10.3109/00498259209049904
